# Safety Profile of Thread-Embedding Acupuncture: A Systematic Review and Meta-Analysis of Randomized Controlled Trials

**DOI:** 10.3390/biomedicines14091901

**Published:** 2026-08-26

**Authors:** Jinyeong Hong, Woo-Hyeon Noh, Jin-woo Suh, Yeonju Woo, Felipe Fregni, Joo-Hee Kim

**Affiliations:** 1 Department of Acupuncture and Moxibustion Medicine, College of Korean Medicine, Sangji University, Wonju 26339, Republic of Korea; hongjy0313@gmail.com; 2 Department of Neuropsychiatry of Korean Medicine, College of Korean Medicine, Sangji University, Wonju 26339, Republic of Korea; 3 Department of Physiology, College of Korean Medicine, Sangji University, Wonju 26339, Republic of Korea; 4 Spaulding Neuromodulation Center, Spaulding Rehabilitation Hospital and Massachusetts General Hospital, Harvard Medical School, Boston, MA 02129, USA; 5 Massachusetts General Hospital, Harvard Medical School, Boston, MA 02114, USA

**Keywords:** thread-embedding acupuncture, suture thread, safety, adverse events, meta-analysis

## Abstract

**Background/Objectives**: Thread-embedding acupuncture (TEA) involves the implantation of suture threads into therapeutic points for sustained stimulation; however, its safety evidence remains limited. This systematic review and meta-analysis evaluated adverse events (AEs) associated with TEA to establish its safety profile. **Methods**: Ten databases were searched up to April 5, 2026 for randomized controlled trials (RCTs) of TEA. Reported AEs were standardized using MedDRA terminology, WHO-UMC causality criteria, and CTCAE version 5.0. Pooled incidence proportions were estimated using generalized linear mixed models, and risk ratios (RRs) were pooled using random-effects models with 95% confidence intervals (CIs). **Results**: Of the 90 RCTs included, most were conducted in China (81, 90.0%). For TEA monotherapy, pooled AE incidence proportion was 3% (95% CI: 2–7%; I^2^ = 53.7%). Local AEs were most common (111, 5.9%), followed by systemic (5, 0.3%) and other AEs (2, 0.1%). Among 118 participants with AEs, most were classified as Grade 1 (95, 80.5%), with Grade 2 (8, 6.8%) and unassessable cases (15, 12.7%). In comparative analyses, TEA showed no significant increase in AE risk versus sham TEA (RR: 1.15, 95% CI: 0.83–1.60), acupuncture (RR: 0.97, 95% CI: 0.64–1.49), or conventional medicine (RR: 0.28, 95% CI: 0.18–0.45). Only one serious TEA-related AE was reported among 1697 participants (0.06%); the withdrawal rate due to TEA-related AEs was 0.36% (9/2524). **Conclusions**: TEA demonstrated a favorable safety profile when used alone or as an add-on therapy, with most AEs mild and transient. However, inconsistencies in AE definitions and reporting highlight the need for standardized terminology and more rigorous safety evaluation in future studies.

## 1. Introduction

Biomaterials interact with host tissues after implantation, eliciting a cascade of immune and inflammatory responses that range from physiological wound healing to foreign body reactions [1]. The nature and extent of these responses depend on the physicochemical properties and degradation of the implanted material, influencing the risk of biomaterial-related adverse events (AEs) such as persistent inflammation, granuloma formation, hypersensitivity, and infection [2,3]. Sutures, the most widely used implantable biomaterials, have long been applied for surgical wound closure and tissue support [4]. As with other biomaterials, their biocompatibility and degradation behavior affect host tissue responses and the occurrence of biomaterial-related AEs [4,5].

Thread-embedding acupuncture (TEA) represents an implantation-based acupuncture technique in which sutures are inserted into therapeutic points to provide prolonged stimulation [6]. Although its mechanisms are not fully understood, experimental studies suggest that TEA may modulate nociceptive pathways and neuroinflammatory responses, while inducing localized aseptic inflammation that promotes collagen synthesis and tissue remodeling [7,8,9,10,11,12,13]. TEA has therefore been applied to diverse conditions, including pain disorders, obesity, allergic diseases, gastrointestinal disorders, and gynecological conditions [14,15,16,17].

Despite its growing clinical use, the safety profile of TEA remains incompletely defined. Conventional acupuncture consistently demonstrates favorable safety outcomes, but these cannot be directly extrapolated to TEA because TEA involves implantation of sutures that remain in tissues after treatment [18,19,20,21]. Similarly, evidence from surgical and cosmetic applications provides insight into biomaterial-related tissue responses, but differences in implantation sites and techniques limit applicability to TEA [22,23,24]. Consequently, TEA requires independent safety evaluation that considers both procedural factors and biomaterial-host interactions.

However, despite the need for independent safety evaluation, evidence regarding the safety of TEA remains limited and is derived primarily from case reports, small-scale studies, and AE data reported as secondary outcomes in randomized controlled trials (RCTs) [25,26,27,28]. Huang et al. conducted the most directly relevant previous systematic review, synthesizing evidence from both RCTs and case reports and providing a broad descriptive overview of TEA-associated AEs [29]. However, design-specific estimates of comparative AE risk across different comparator interventions, together with standardized reclassification of AE terminology, causality, and severity, remained limited. In addition, inconsistency in AE reporting and classification—due to non-standardized definitions and reliance on subjective assessment—has led to variability in causality and severity classification, reducing comparability and contributing to heterogeneity in pooled evidence [30,31]. This methodological inconsistency may limit the interpretability of TEA safety outcomes and highlights the need for more standardized and comparative evidence to support clinical decision-making [32]. Building on this evidence base and the prospectively published protocol for the present review [33], we systematically re-evaluated AEs reported in RCTs using harmonized terminology and standardized causality and severity criteria and conducted comparative meta-analyses across control interventions.

## 2. Materials and Methods

The protocol for this SR was prospectively registered in the International Prospective Register of Systematic Reviews (PROSPERO) (CRD42022297123) and published as a protocol [33], and the study was conducted in accordance with the PRISMA 2020 statement (Table S1) and PRISMA-harms checklist (Table S2) [34,35]. The protocol prespecified the inclusion of both RCTs and non-randomized (observational) studies evaluating the safety of TEA. After completion of full-text screening and final study selection, but before data extraction, the review team decided to analyze randomized and non-randomized evidence separately because of methodological differences in risk-of-bias assessment and data synthesis. Accordingly, the present review was restricted to RCTs, and evidence from observational studies will be reported in a companion SR (in preparation) derived from the same protocol. This amendment was recorded in PROSPERO on 26 June 2026.

### 2.1. Search Strategy

We comprehensively searched 10 international and regional databases for RCTs utilizing TEA, including studies published up to April 5, 2026. The databases included PubMed, Embase, the Cochrane Library, Web of Science, China National Knowledge Infrastructure (CNKI), Citation Information by National Institute of Informatics (CiNii), Japan Science and Technology Information Aggregator, Electronic (J-STAGE), Korean Studies Information Service System (KISS), ScienceON, and Oriental Medicine Advanced Searching Integrated System (OASIS). The search terms combined Medical Subject Headings, Emtree terms, and relevant free-text terms with Boolean operators. Searches were conducted in English and in languages supported by each database, including Chinese, Japanese, and Korean. The search included the gray literature indexed in these sources, such as dissertations, conference abstracts, and conference proceedings. Backward citation searching of included studies and relevant reviews was also conducted. For trial protocols or registry-linked records identified during screening, the corresponding registry entries were examined for linked publications, publicly posted results, and available AE or SAE data. The detailed search strategies for each database are provided in Table S3.

### 2.2. Eligibility Criteria

For the safety assessment of TEA, participants of any age, sex, race, baseline comorbidities, or target diseases were eligible, and no restrictions were imposed on the publication language, status, or format. Studies comparing TEA as monotherapy with TEA or other interventions, or as an add-on therapy versus the same co-intervention alone, were included. Eligibility for the systematic review and analyses of all reported AEs did not depend on causal attribution. Studies were eligible when AE data were collected and reported for all relevant study arms. Studies were excluded if AEs were not collected, were collected in only one study arm, or were collected only for a specific intervention among the included interventions. To minimize data loss, studies with single-zero and double-zero events were included. The primary outcome was the participant-level incidence proportion of TEA-related AEs. Secondary outcomes included the characteristics of reported AEs (type, severity, duration), participant-level comparative safety across interventions, incidence of serious adverse events (SAEs), and withdrawals due to AEs. Prespecified subgroup analyses were conducted by AE reporting method and thread material.

### 2.3. Study Selection and Data Extraction

The screening process was conducted independently by two researchers (J.H. and W-H.N.), and any disagreements were resolved through consensus with a third researcher (J-H.K.). After removing duplicate records, titles and abstracts were screened against eligibility criteria. Full texts of potentially eligible studies were then examined, and exclusions were made based on the eligibility criteria. Additional studies identified through citation searching were assessed using the same eligibility criteria. References were managed using EndNote 21 software. For studies included in the final analysis, the following data were extracted: first author’s affiliation, year and country of publication, number of participants, target disease, interventions applied, types of threads used, treatment frequency, treatment duration, and any reported AEs.

### 2.4. Risk of Bias and Harms-Reporting Quality Assessment

Risk of bias was assessed using the Cochrane Risk of Bias 2.0 (RoB 2.0) tool, which evaluates five domains: (1) randomization process, (2) deviations from intended interventions, (3) missing outcome data, (4) outcome measurement, and (5) selection of the reported result. Each domain was rated as low risk, some concerns, or high risk. Although Domain 4 of RoB 2.0 is typically applied to efficacy outcomes, it was used here to evaluate bias in AE assessment and reporting, consistent with the study’s focus on TEA safety. Overall risk of bias was determined by the highest rating across domains, classified as “some concerns” if any domain had some concerns, and “high risk” if any domain was rated high risk.

As the RoB 2.0 tool alone has limitations in evaluating the quality of AE reporting, the McMaster Tool for Assessing Quality of Harms Assessment and Reporting in Study Reports (McHarm) was additionally applied [36,37]. The McHarm tool consists of 15 items rated as Yes, No, or Unclear based on information reported in the original publication, study protocol, or trial registry. A study was classified as “Yes” when sufficient information was reported to satisfy the assessment criteria, “No” when the reported information was insufficient to satisfy the criteria, and “Unclear” only when the available data did not permit a definitive assessment.

### 2.5. Standardization of Adverse-Event Terminology

To minimize distortion caused by heterogeneous terminology and to ensure accuracy and reproducibility, the Medical Dictionary for Regulatory Activities (MedDRA) version 29.0, developed by the International Council for Harmonization of Technical Requirements for Pharmaceuticals for Human Use (ICH), was used to systematically group reported AE terms [38]. Each AE term was categorized under a Preferred Term (PT), which served as the representative term in the meta-analysis. Clinically similar reactions reported using heterogeneous terminology were mapped separately but analyzed as a single category for frequency analysis. Symptoms considered secondary manifestations of broader AEs, such as chills associated with infection, were not analyzed independently. In contrast, reactions that may co-occur but also occur independently were treated as separate AEs. Terms with potential ambiguity due to translation or reporting differences were standardized to terminology most commonly used in the relevant clinical context. Detailed MedDRA PT mappings are provided in Table S4.

### 2.6. Causality Assessment

We used the World Health Organization-Uppsala Monitoring Centre (WHO-UMC) assessment criteria, a tool that evaluates causality based on the relationship between timing, response to withdrawal, and re-challenge, classifying events as certain, probable/likely, possible, unlikely, conditional/unclassified, or unassessable/unclassifiable [39]. We considered the minimum level of causality for AEs as “possible”.

“Possible” was defined as an AE with a reasonable temporal relationship to the intervention that could also be explained by other causes. Systemic reactions associated with non-pharmacological interventions were considered temporally related when they occurred during or immediately after the procedure. Local reactions were primarily evaluated based on their relationship to the treatment site, considering intervention characteristics and contextual factors. “Probable/likely” required, in addition to “possible” criteria, a clinically reasonable response to withdrawal when applicable, and exclusion of disease or other interventions as causes. “Certain” required supportive evidence from re-challenge when available, along with a clear clinical or pharmacological pattern. AEs were classified as “unlikely” when temporal or spatial association was weak and causality was unsupported. Cases with insufficient information were classified as “unassessable”.

### 2.7. Severity Assessment

AE severity was assessed using the Common Terminology Criteria for Adverse Events (CTCAE) version 5.0, which establishes CTCAE Terms based on MedDRA PTs and provides grading criteria [40]. Grade 1 included mild symptoms that resolved spontaneously or required only local conservative therapy (e.g., hot packs, infrared therapy) without affecting activities of daily living. Grade 2 included moderate AEs requiring active treatment such as oral or topical medication, or clinically significant self-limiting moderate symptoms. Grade 3 included severe AEs requiring invasive procedures or hospitalization. Grade 4 included life-threatening consequences requiring urgent intervention, and Grade 5 indicated death related to an AE. SAEs were defined according to the ICH E2A guideline, with unrelated surgical or hospitalization events reclassified accordingly.

MedDRA terminology mapping, WHO-UMC causality assessment, and CTCAE severity grading were independently performed by two reviewers (J.H. and W-H.N.) based on the information reported in the original studies. Disagreements were resolved through consensus with a third reviewer (J-H.K.).

### 2.8. Statistical Analysis

For the causality-specific analysis of TEA-related AEs, TEA monotherapy arms were included when the number of participants experiencing at least one AE classified as certain, probable/likely, or possible according to the WHO-UMC criteria could be determined. Studies were excluded from this analysis only when the participant-level numerator could not be ascertained because of insufficient causality information. All incidence and comparative analyses were based on the number of participants experiencing at least one AE, rather than the total number of AE episodes. Pooled incidence proportions with corresponding 95% confidence intervals (CIs) were estimated using a generalized linear mixed model, which was selected to appropriately model rare and zero-event incidence data without relying on continuity corrections [41]. The crude aggregate proportion was calculated descriptively by dividing the total number of participants experiencing at least one AE by the total number of participants assessed across studies and was not used as the meta-analytic summary estimate. Subgroup and sensitivity analyses were conducted to investigate potential sources of heterogeneity.

To evaluate differences in overall AE risk between intervention groups, all reported AEs were included regardless of their causality assessment, and pooled risk ratios (RRs) with 95% CIs were estimated using the Mantel–Haenszel method, whereas pooled risk differences (RDs) were estimated using the inverse-variance method under a random-effects framework. For comparative analyses involving zero cells, a continuity correction of 0.5 was applied to studies containing at least one zero cell. Between-study variance was estimated using the DerSimonian–Laird method, and the Hartung–Knapp adjustment was applied to CIs and hypothesis testing. Heterogeneity was assessed by I^2^, with 0–40% considered not important, 30–60% moderate, 50–90% substantial, and 75–100% considerable heterogeneity [42,43]. A zero-event count was assigned only when a study explicitly stated that no AEs occurred or provided an arm-level zero count following AE assessment. Absence of AE reporting was classified as not reported rather than as zero. To assess the robustness of the comparative estimates to the handling of sparse events, sensitivity analyses were performed after excluding double-zero-event studies.

Inter-rater agreement for each assessment was calculated from the pre-consensus ratings using unweighted Cohen’s k and Gwet’s Agreement Coefficient 1 (AC1) [44,45]. Cohen’s k was interpreted as follows: <0.00, poor; 0.00–0.20, slight; 0.21–0.40, fair; 0.41–0.60, moderate; 0.61–0.80, substantial; and 0.81–1.00, almost perfect agreement [46].

Selective reporting and small-study effects were assessed by examining funnel plot asymmetry when ≥10 studies were available, with Egger’s regression test applied for statistical evaluation. All statistical analyses were performed using R (version 4.5.2; R Foundation for Statistical Computing, Vienna, Austria). All results were visually presented using a forest plot.

## 3. Results

### 3.1. Characteristics of Included Studies

**Figure 1:**
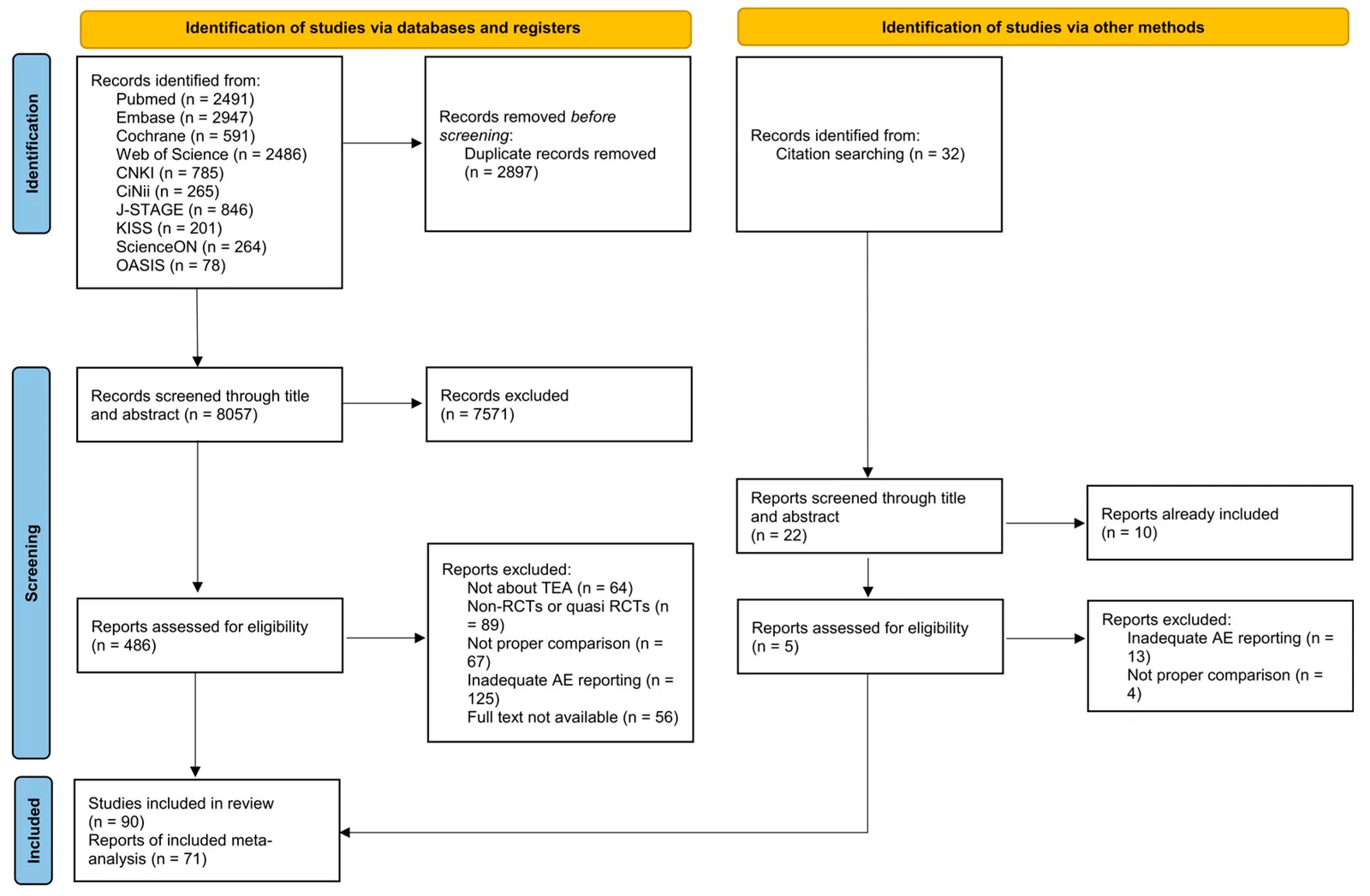
PRISMA flow chart.

Of 10,954 identified records, 2897 duplicates were removed, leaving 8057 studies for title and abstract screening. Through the first screening stage, 486 articles underwent full-text review. Following full-text assessment based on the pre-defined eligibility criteria, 85 studies were included, with five additional studies identified through citation searching. In total, 90 studies [47,48,49,50,51,52,53,54,55,56,57,58,59,60,61,62,63,64,65,66,67,68,69,70,71,72,73,74,75,76,77,78,79,80,81,82,83,84,85,86,87,88,89,90,91,92,93,94,95,96,97,98,99,100,101,102,103,104,105,106,107,108,109,110,111,112,113,114,115,116,117,118,119,120,121,122,123,124,125,126,127,128,129,130,131,132,133,134,135,136] were included in the SR, and 71 studies supported by at least two comparisons were included in the meta-analysis (Figure 1).

Most studies originated from China (81, 90.0%), followed by Korea (seven, 7.8%), with one study each from Vietnam and Denmark. After excluding 12 studies that did not clearly report thread material and three that compared different thread materials, 75 studies remained. Among these, catgut was most frequently used (47, 62.7%), followed by polydioxanone (PDO) 13 (17.3%), poly (glycolide-co-lactide) (PGLA) (11, 14.7%), polyglycolic acid (PGA) (3, 4.0%), and gold thread (one, 1.3%). Studies in Korea (n = 7) and Vietnam (n = 1) used only PDO, while the single study from Denmark used gold thread. Catgut, PGLA, and PGA were exclusively used in studies conducted in China. Among the 68 studies reporting needle diameter (excluding 22 that did not provide this information), 0.7 mm was most commonly used (29, 42.6%), followed by 0.9 mm (14, 20.6%), and 0.8 mm (7, 10.3%). The average interval between treatment sessions in 82 studies was 11.7 days, with 14–15 days being the most frequently reported (36, 43.9%), followed by 1 week (28, 34.1%) and 10 days (14, 17.1%). The average number of treatment sessions was 5.0; four sessions was the most frequently reported (20, 24.4%), followed by six (16, 19.5%) and three (15, 18.3%) (Table S5).

### 3.2. Risk of TEA-Related AEs

All analyses were based on participants who received at least one TEA session, and only studies reporting total participant numbers were included in the meta-analysis. Studies were considered to have zero AEs only when they explicitly identified the AEs targeted for collection. A total of 1883 participants from 33 studies [47,48,49,50,51,52,53,54,55,56,57,58,59,60,61,62,63,64,65,66,67,68,69,70,71,72,73,74,75,76,77,78,79] were analyzed. The pooled incidence proportion of participants experiencing at least one AE was 3% (95% CI: 2% to 7%; I^2^ = 53.7%). Across studies, the crude incidence proportion was 6.3% (118/1883). The crude estimate was calculated by pooling the numbers of affected participants and participants assessed across studies, whereas the GLMM synthesized study-specific proportions while accounting for between-study heterogeneity. Because these approaches use different weighting structures and estimate different quantities, the resulting values are not expected to be identical.

**Figure 2:**
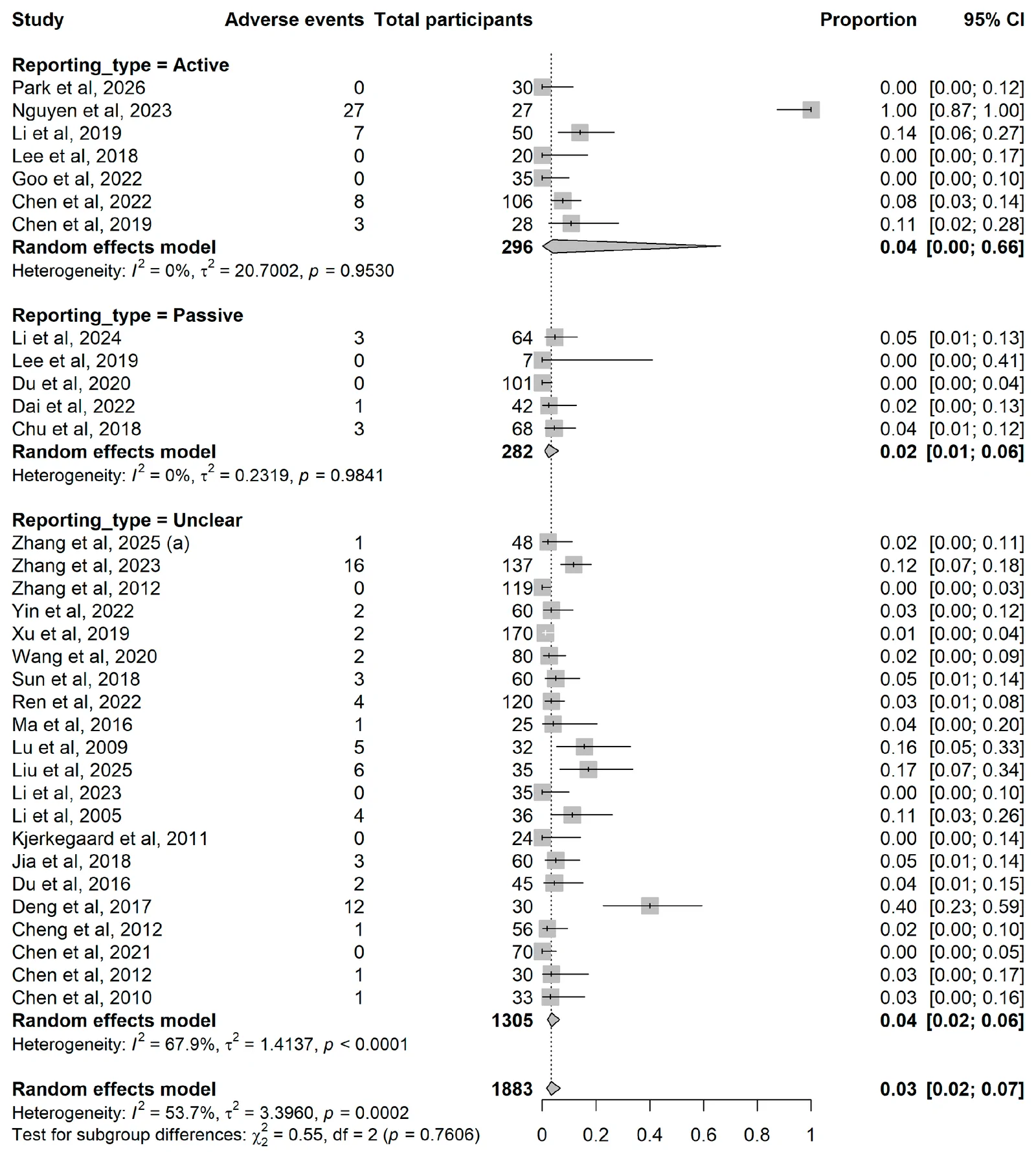
Forest plot of the pooled incidence of thread-embedding-acupuncture-related adverse events according to reporting type [47,48,49,50,51,52,53,54,55,56,57,58,59,60,61,62,63,64,65,66,67,68,69,70,71,72,73,74,75,76,77,78,79].

To assess potential influence of underreporting on AE incidence proportion, studies were classified into active, passive, and unclear reporting groups. RoB assessment indicated fewer high-risk studies in the active (0%, 0/7) and passive (40%, 2/5) reporting groups compared with the unclear reporting group (81.0%, 17/21). McHarm assessment similarly indicated higher reporting quality in active and passive reporting groups than in the unclear reporting group. Despite differences in methodological and reporting quality, AE incidence proportions were broadly comparable across reporting types (*p* = 0.76): active reporting 4% (95% CI: 0% to 66%; I^2^ = 0%), passive reporting 2% (95% CI: 1% to 6%; I^2^ = 0%), and unclear reporting 3% (95% CI: 2% to 6%; I^2^ = 67.9%) (Figure 2).

For analysis according to AE type, participants could contribute to multiple AE categories, but repeated occurrences of the same AE type were counted only once per participant. Among 1883 participants, local AEs were reported in 111 (5.9%), systemic AEs in five (0.3%), and other AEs in two (0.1%). Among all AEs, pain (pooled incidence 6%, 95%: CI 0% to 48%; I^2^ = 0%), nodule/induration (pooled incidence 4%, 95% CI 1% to 9%; I^2^ = 77.5%), and hemorrhage (pooled incidence 4%, 95% CI 2% to 9%; I^2^ = 47.5%) were the most frequently estimated AEs, whereas other AEs were uncommon.

Regarding severity, AEs were classified as Grade 1 in 95 participants (80.5%), Grade 2 in eight (6.8%), and unassessable in 15 (12.7%). Of the eight participants with Grade 2 AEs, six experienced local AEs and two experienced systemic AEs. Local Grade 2 AEs included pain (n = 2), swelling with erythema (n = 2), and allergic reactions (n = 2). Swelling/erythema and allergic reactions required topical erythromycin ointment and antihistamines, respectively, and resolved. Management and outcomes were not reported for the two cases of pain. The two systemic Grade 2 AEs were pre-syncope; one resolved with usual care, while the outcome of the other was not reported.

Symptom duration was not reported for 56 of the 118 participants with AEs. Among the remaining 62, 58 (93.5%) experienced resolutions within 7 days, whereas four experienced relatively prolonged AEs. One participant had nodule/induration that resolved after 2 weeks but was accompanied by hyperpigmentation persisting for 2 months. Three participants experienced nodule/induration lasting 1–3 months. No long-term AEs were reported in studies with follow-up of ≥3 months, including those with ≥6 months of follow-up. Details are summarized in Table 1.

**Figure 3:**
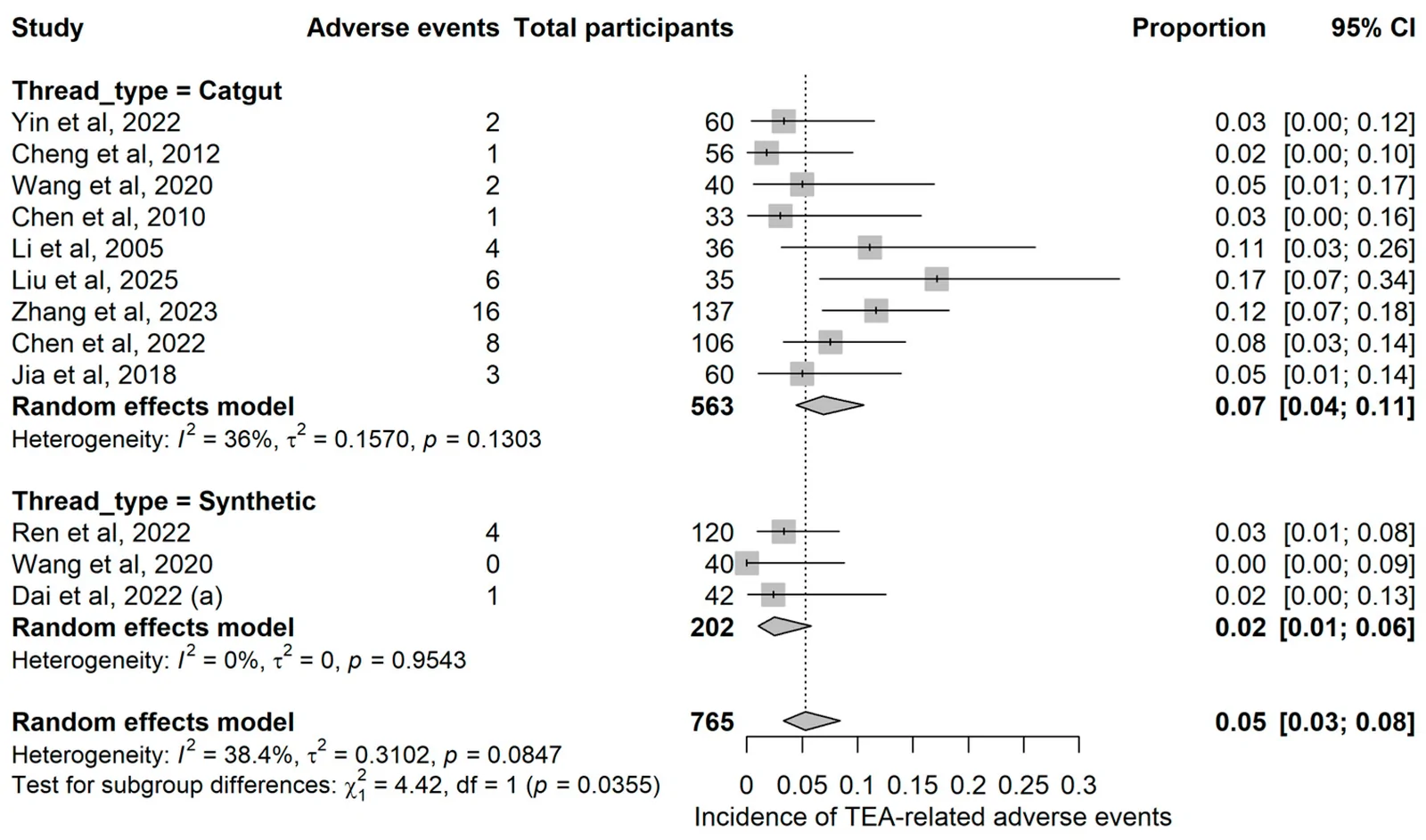
Forest plot of the pooled incidence of thread-embedding-acupuncture-related adverse events according to thread type [48,50,51,55,57,65,67,74,75,78,79].

An exploratory subgroup analysis was conducted to evaluate the potential influence of thread material on AE incidence proportion using studies with comparable treatment conditions (acupoint location, treatment interval, and needle gauge). Twelve arms from 11 studies, including one head-to-head comparison of catgut and synthetic thread, were analyzed. The pooled incidence proportion was 7% (95% CI 4% to 11%; I^2^ = 36%) in the catgut group and 2% (95% CI 1% to 6%; I^2^ = 0%) in the synthetic thread group, with a statistically significant between-subgroup difference (*p* = 0.04). This difference was primarily attributable to local AEs, particularly nodules/induration, which were reported in 20 (3.6%) participants in the catgut group compared with one (0.5%) in the synthetic thread group. Allergic reactions and infections were reported only in the catgut group, with two cases each (Figure 3).

### 3.3. Risk of Overall AEs

A total of 90 studies were identified, including 52 head-to-head comparison studies and 32 add-on therapy studies. Among these, only studies reporting the total number of participants were included in the meta-analysis, resulting in 43 head-to-head comparison studies [55,56,57,58,59,60,61,62,63,64,65,66,67,68,69,70,71,72,73,74,75,76,77,78,79,80,81,82,83,84,85,86,87,88,89,90,91,92,93,94,95,96,97] and 23 add-on therapy studies [95,96,97,98,99,100,101,102,103,104,105,106,107,108,109,110,111,112,113,114,115,116,117].

#### 3.3.1. Head-to-Head Comparison

**Figure 4:**
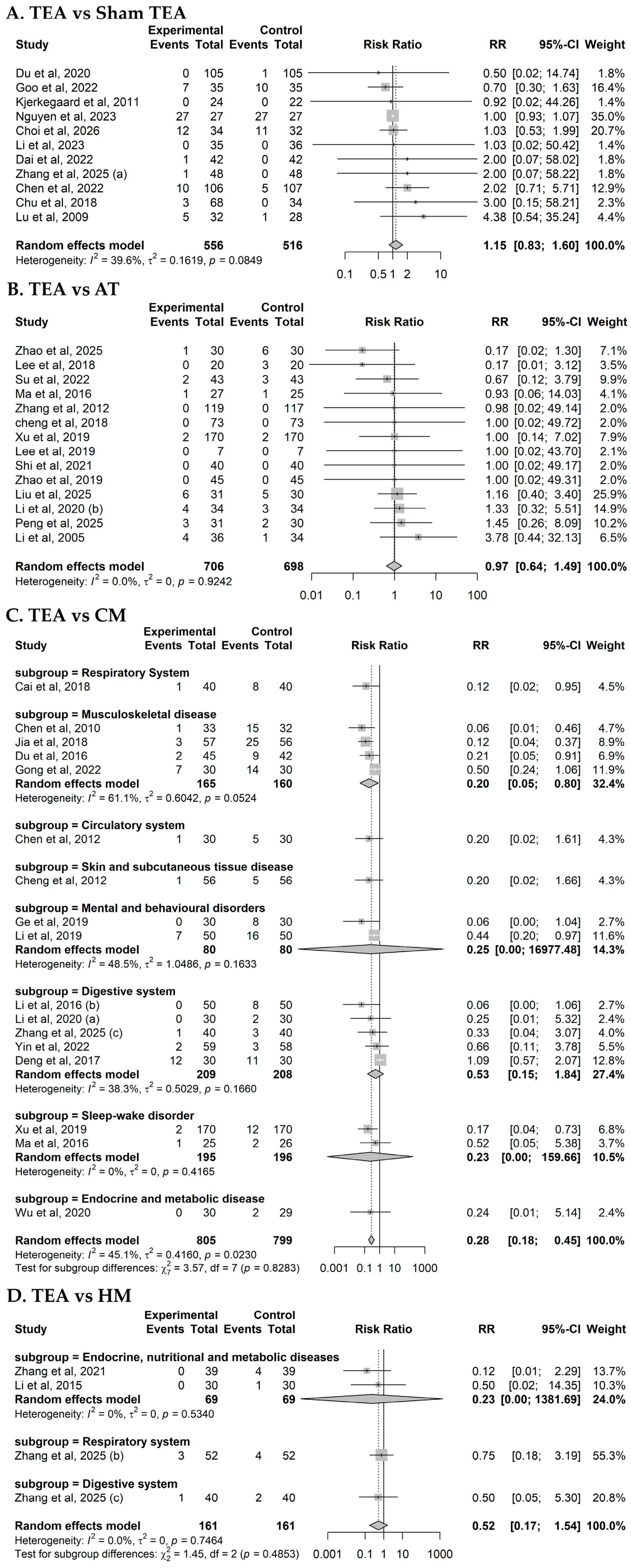
Forest plot of risk ratios for overall adverse events in head-to-head comparisons (including double zero event studies) [55,56,57,58,59,60,61,62,63,64,65,66,67,68,69,70,71,72,73,74,75,76,77,78,79,80,81,82,83,84,85,86,87,88,89,90,91,92,93,94,95,96,97].

Sham TEA was defined as a procedure identical to TEA in treatment site, interval, and needle manipulation, except that no thread was inserted. Compared with sham TEA, AE risk did not differ significantly (11 studies, TEA 66/556 vs. Sham TEA 55/516; RR: 1.15, 95% CI: 0.83 to 1.60, I^2^ = 39.6%; RD: 0.01, 95% CI: −0.01 to 0.02, I^2^ = 0%). Compared with acupuncture treatment (AT), AE risk was also not significantly different (14 studies, TEA 23/706 vs. AT 26/698; RR: 0.97, 95% CI: 0.64 to 1.49, I^2^ = 0%; RD: 0.00, 95% CI: −0.01 to 0.01, I^2^ = 0%). TEA was associated with lower risk reported AEs than conventional medicine (CM) (17 studies, TEA 41/805 vs. CM 148/799; RR: 0.28, 95% CI: 0.18 to 0.45, I^2^ = 45.1%; RD: −0.13, 95% CI: −0.19 to −0.07, I^2^ = 69.2%), whereas no significant difference was observed compared with herbal medicine (HM) (four studies, TEA 4/161 vs. HM 11/161; RR: 0.52, 95% CI: 0.17 to 1.54; I^2^ = 0%; RD: −0.04, 95% CI: −0.10 to 0.02; I^2^ = 0%) (Figure 4 and Figure S1).

#### 3.3.2. Add-On Therapy Comparison

**Figure 5:**
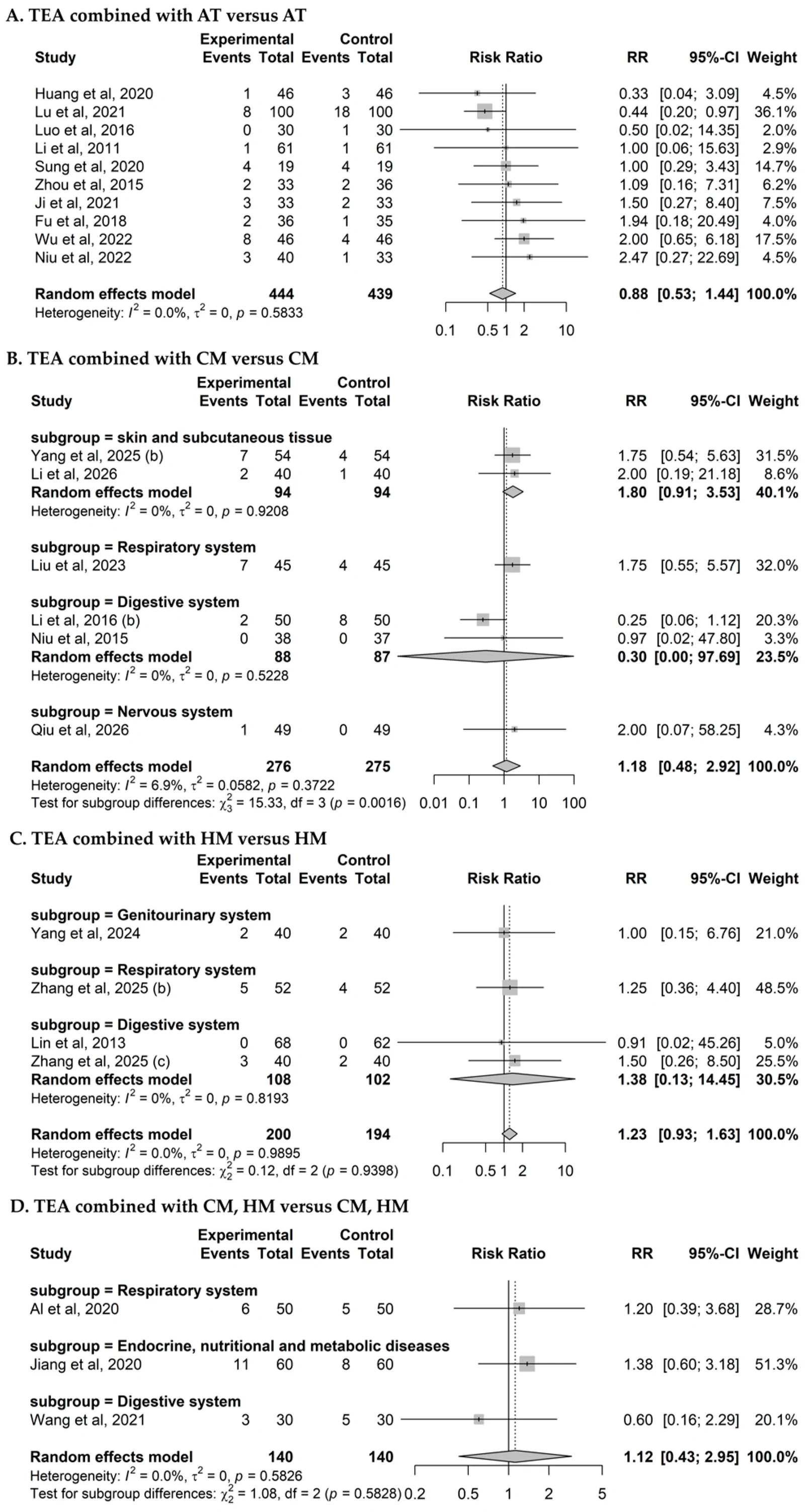
Forest plot of the risk ratio of overall adverse events in studies comparing add-on therapy with thread-embedding acupuncture (including double zero event studies) [95,96,97,98,99,100,101,102,103,104,105,106,107,108,109,110,111,112,113,114,115,116,117].

AT add-on therapy showed no significant difference in AE risk between groups (10 studies, TEA+AT 32/444 vs. AT 37/439; RR: 0.88, 95% CI: 0.53 to 1.44, I^2^ = 0%; RD: −0.01, 95% CI: −0.04 to 0.03, I^2^ = 0%). As add-on therapy to pharmacological treatment, no significant differences in AE risk were observed for either CM (six studies, TEA+CM 19/276 vs. CM 17/275; RR: 1.18, 95% CI: 0.48 to 2.92, I^2^ = 6.9%; RD: 0.01, 95% CI: −0.04 to 0.06, I^2^ = 24.2%) or HM (four studies, TEA+HM 10/200 vs. HM 8/194; RR: 1.23, 95% CI: 0.93 to 1.63, I^2^ = 0%; RD: 0.00, 95% CI: −0.01 to 0.02, I^2^ = 0%). Even when CM and HM were combined, adding TEA did not significantly alter AE risk (three studies, TEA+CM+HM 20/140 vs. CM+HM 18/140; RR: 1.12, 95% CI: 0.43 to 2.95, I^2^ = 0%; RD: 0.01, 95% CI: −0.12 to 0.14; I^2^ = 0%) (Figure 5 and Figure S2).

### 3.4. Risk of Intervention-Related AEs

Among the studies included in the overall risk analysis, those without AEs classified as unassessable in the causality assessment were analyzed for intervention-related AE risk, defined as AEs with causality of at least “possible”. Of these, 19 studies [54,55,56,57,58,59,60,61,62,63,64,65,66,67,68,69,70,71,88] were monotherapy comparisons and 10 studies [98,99,100,101,102,103,104,105,106,107] were add-on therapy comparisons.

#### 3.4.1. Head-to-Head Comparison

Sham TEA showed no significant difference in AE risk compared with TEA (10 studies, TEA 40/552 vs. Sham TEA 32/488; RR: 1.30, 95% CI: 0.998 to 1.69; I^2^ = 39.5%; RD: 0.01, 95% CI: −0.00 to 0.01; I^2^ = 0%). AT-controlled studies also showed no significant difference (seven studies, TEA 13/331 vs. AT 11/326; RR: 1.16, 95% CI: 0.65 to 2.07; I^2^ = 0%; RD: 0.00, 95% CI: −0.02 to 0.02; I^2^ = 0%). In contrast, CM-controlled studies showed no significant difference AE risk between groups with wide CI (three studies, TEA 16/245 vs. CM 32/242; RR: 0.38, 95% CI: 0.03 to 5.32; I^2^ = 78.4%; RD: −0.08, 95% CI: −0.24 to 0.09; I^2^ = 31.4%) (Figure S3 and Figure S4). In the HM-controlled study, AEs were reported only in the HM group.

#### 3.4.2. Add-On Therapy Comparison

TEA combined with acupuncture therapy (TEA+AT) showed no significant difference in AE incidence between groups (seven studies, TEA+AT 15/275 vs. AT 11/271; RR: 1.39, 95% CI: 0.82 to 2.36; I^2^ = 0%; RD: 0.00, 95% CI: −0.03 to 0.03; I^2^ = 0%) (Figure S5). Among pharmacological add-on studies with causality assessment, three were included: two combining TEA with CM [101,105] and one combining TEA with HM [99]. Of the two TEA+CM studies, one reported no AEs in either group, whereas the other reported a single local AE in the TEA group. In the TEA+HM study, AEs were reported in 5.0% (2/40) of participants in both groups; redness and irritation were reported in the TEA group, whereas diarrhea was reported in the HM group.

### 3.5. Sensitivity Analysis

Sensitivity analyses excluding double-zero event studies showed consistent results across all comparisons, with no evidence of publication bias (Figures S6 and S7). In head-to-head analyses, heterogeneity decreased after excluding transient AEs reported by Nguyen et al. [68], in which mild pain occurred uniformly in both groups and resolved within 15 min (10 studies, TEA 17/522 vs. Sham TEA 8/488; RR: 1.50, 95% CI: 1.23 to 1.83, I^2^ = 0%; RD: 0.01, 95% CI: −0.00 to 0.02, I^2^ = 0%). Excluding the study by Deng et al. [58], which reported an unusually high incidence of AEs following perineal-area treatment, there was also decreased heterogeneity (16 studies, TEA 29/775 vs. CM 137/769; RR: 0.28, 95% CI: 0.19 to 0.41, I^2^ = 0%) (Figures S8 and S9). While heterogeneity decreased, RR estimates varied slightly, whereas RD estimates remained stable. In CM add-on therapy analyses, exclusion of an influential study (Li et al. [96]) resulted in some variation in RR estimates, whereas RD estimates remained unchanged (five studies, TEA+CM 17/226 vs. CM 9/225; RR: 1.75, 95% CI: 1.47 to 2.07, I^2^ = 0%; RD: 0.02, 95% CI: −0.01 to 0.05, I^2^ = 0%) (Figure S10).

### 3.6. Serious Adverse Events

Among the 35 studies reporting SAEs, the crude incidence proportion was 0.06% (1/1697) in TEA groups and 0.07% (1/1535) in control groups. Only one TEA-related SAE was identified, an iatrogenic pneumothorax occurring during thoracic TEA in a participant with non-specific neck pain, attributed to procedural error. The participant fully recovered after 16 days of inpatient and outpatient management. In the control groups, one case of acute cholecystitis was reclassified as an SAE based on severity criteria but was considered unrelated to the intervention.

### 3.7. Withdrawal

Among 46 studies reporting participant withdrawals, the overall withdrawal rate was 5.3% (133/2524) in TEA groups. Withdrawals attributable to AEs occurred in 14 participants (0.55%), of which nine (0.36%) were considered TEA-related. Reported reasons included TEA-related pain (n = 6), pneumothorax (n = 1), infection at the thread-embedding site (n = 2), and allergic reactions attributed to concomitant medications (n = 5). In control groups, the overall withdrawal rate was 5.6% (115/2066), with eight participants (0.39%) withdrawing due to AEs, including medication-related AEs (n = 5) and AT-related pain (n = 3).

### 3.8. Quality of Studies

The McHarm tool includes domains relevant to incidence analyses, including AE pre-definition (Q1–3), reporting type (Q5–6), and quantitative reporting (Q12–14). Overall, reporting was limited in the pre-definition and reporting type domains, whereas quantitative reporting was relatively well documented. In the pre-definition domain, most studies reported the types of AEs collected; however, definitions, classification criteria, and severity grading systems varied across studies, suggesting the absence of a standardized reporting framework for TEA-related AEs. In the reporting type domain, most studies stated that AEs were monitored during the study period, but detailed descriptions of AE collection and assessment procedures were inconsistently reported. In contrast, quantitative AE reporting was generally adequate. Most studies provided data on either the number of participants experiencing AEs and/or the total number of AEs. Except for a small number of studies in which it was unclear whether AE counts referred to events or participants, the data were sufficient for quantitative synthesis (Table S6).

RoB assessment showed one study at high risk in Domain 1 due to baseline imbalance between groups. In Domain 2, 85 studies (94.4%) were rated as having some concerns due to insufficient information on deviations from intended interventions, but none were high risk. In Domain 3, 59 studies (65.6%) were rated as having some concerns due to incomplete reporting of missing outcome data, and three studies were high risk due to imbalances in missing participants or reasons for missing data. In Domain 4, 66 studies (73.3%) were rated high risk and 14 as having some concerns, mainly due to limitations in outcome measurement and lack of blinding in AE assessment inherent to open-label TEA designs. In Domain 5, 81 studies (90.0%) were rated as having some concerns due to absence of prespecified protocols or statistical analysis plans, and no study was rated as high risk (Figure S11).

### 3.9. Inter-Rater Reliability

Inter-rater agreement was almost perfect for causality assessment (unweighted Cohen’s k = 0.86, 95% CI 0.60–1.00; Gwet’s AC1 = 0.90, 95% CI 0.81–1.00), severity assessment (unweighted Cohen’s k = 0.94, 95% CI 0.73–1.00; Gwet’s AC1 = 0.95, 95% CI 0.88–1.00), and MedDRA terminology mapping (unweighted Cohen’s k = 0.87, 95% CI 0.81–0.92; Gwet’s AC1 = 0.87, 95% CI 0.78–0.96).

## 4. Discussion

### 4.1. Major Findings

This study systematically synthesized AEs reported in 90 RCTs using consistent assessment criteria to evaluate the safety of TEA. The pooled incidence proportion of TEA-related AEs was 3% (95% CI: 2% to 7%; I^2^ = 53.7%).

Most reported AEs were mild local reactions, most commonly nodule/induration, pain, and hemorrhage. Where the clinical course was reported, these events generally resolved spontaneously or with simple conservative management within a few days. Comparative analyses showed TEA did not consistently increase AE risk compared with sham TEA or active controls. Although sensitivity analyses revealed some variability in relative effect estimates, absolute difference estimates remained stable. This discrepancy likely reflects the low incidence of AEs, where small absolute differences may appear amplified in relative terms [137,138,139]. Overall, the available RCTs did not show a consistent increase in reported AE risk with TEA across the evaluated comparisons. However, these findings apply to AEs documented under randomized trial conditions and do not exclude uncommon, delayed, or operator-related complications.

The present findings are consistent with a previous SR by Huang et al. [29], who reported induration, pain, hemorrhage, and swelling as the most common AEs. Although not included in the present review, two cohort studies [140,141] similarly identified pain and hemorrhage as the predominant AEs, which were generally transient and mild. Similarly, in an observational study by Ha et al. [26], AEs were predominantly mild, early-onset pain and hemorrhage. Notably, neither the 6-month follow-up study by Ha et al. [26] nor that by Geng et al. [140] reported delayed AEs. Although generally mild, transient, and consistent with expected local responses to needling or thread implantation, these events nonetheless qualify as AEs; their clinical relevance should be judged by severity, duration, management requirements, and outcomes.

Although the overall AE risk of TEA was comparable to that of non-thread needling interventions (sham TEA and AT), differences in AE patterns were observed. Notably, nodule/induration was reported only in the TEA groups and was absent in sham TEA and AT groups. These findings align with prior evidence identifying nodule/induration as the most frequent local AE following TEA, suggesting this distinctive AE pattern is related to implanted threads rather than needling alone [29]. Also, Wang et al. [141] and several case reports [142,143,144] have described rare case of induration or nodules and foreign-body reactions that persisted for several months. However, nodule formation, induration, and foreign-body reactions have not been identified as characteristic AEs in reports of conventional acupuncture safety [19]. As TEA involves biomaterial implantation, these local reactions likely reflect physiological tissue responses associated with biomaterial implantation. These responses may be influenced by thread material, implantation site, and patient characteristics.

Thread material has been suggested as a determinant of local tissue reactions, with catgut associated with a relatively higher risk of AEs compared with synthetic threads [29]. In this study, a similar pattern was observed, with differences in pooled AE incidence and AE types between catgut and synthetic threads, although these findings were not derived from direct comparisons. These differences may be attributed to distinct degradation mechanisms: catgut is degraded via macrophage-mediated phagocytosis and enzymatic digestion, whereas synthetic threads such as PDO/PGA are primarily degraded through hydrolysis, producing more predictable and milder tissue reactions [145,146,147,148]. During this degradation process, local reactions such as nodule/induration, or foreign-body reaction may occur [8,10,149,150,151]. Although these reactions may reflect responses to thread-related tissue reaction, their occurrence as distinctive AEs associated with TEA, rather than conventional acupuncture, warrants careful monitoring and appropriate management [2,152].

In addition to thread material, anatomical factors related to the treatment site may contribute to variability in TEA safety. Differences in vascularity, innervation, and tissue characteristics can influence both mechanical injury and inflammatory response [1,3]. Several studies reported relatively high incidences of nodule/induration at specific treatment sites, including the perineum, Yingxiang, and Yintang acupoints [52,58,132]. These findings suggest possible site-specific variation; however, insufficient reporting on treatment locations and AE characteristics limited further evaluation.

The present findings have important clinical implications for safe TEA application. Multiple factors including thread material, treatment site, patient characteristics, and procedural technique should be considered when planning treatment. Needling in anatomically vulnerable regions is closely associated with operator proficiency. Moreover, AEs such as iatrogenic pneumothorax have been reported with conventional acupuncture [49,153,154,155]. Consistent with these data, the only pneumothorax identified in the present review was attributed to procedural error [124], while a separate case report similarly described neuralgia resulting from a procedural error during cervical TEA treatment [156]. Although not identified in the present review, granuloma and infectious abscesses have been described in case reports, including cases occurring in unregulated medical settings [144,157,158]. Accordingly, adequate operator training, careful adherence to procedural safety principles, and patient education are essential to minimize procedure-related AEs. Although rare, transient pre-syncope may occur during or shortly after treatment, and appropriate peri-procedural and long-term monitoring should be considered to detect both immediate and delayed AEs.

### 4.2. Reporting Quality and Methodological Heterogeneity

Safety-related interventional studies consistently demonstrate methodological issues in AE collection and reporting, particularly the absence of standardized AE definitions and assessment methods, which has been identified as a major source of heterogeneity in safety reporting [43,159,160,161]. In addition, passive reporting systems have been associated with lower detection rates of AEs, raising concerns regarding potential underestimation of AE incidence [162,163]. Collectively, these methodological issues may affect the completeness and consistency of AE reporting and limit comparability across studies [164].

In the present study, similar patterns were observed in the included trials. The quality of AE reporting was assessed using the McHarm and RoB 2.0 tools, which identified important limitations in AE definitions, terminology, and reporting procedures. Clinically similar reactions were not always described or classified consistently across studies. For example, local tissue reactions were variably reported as “induration” or “nodule,” and transient vasovagal reactions were sometimes classified as syncope despite the absence of documented loss of consciousness [51,52,53,65,68,124]. Such variability may have affected the consistency and comparability of pooled estimates. Although studies with explicitly asymmetric arm-level AE collection were excluded, incomplete reporting of AE definitions and ascertainment procedures prevented us from fully excluding residual differences in AE detection between intervention groups, particularly in comparisons with conventional medicine. Overall, these findings highlight the need for standardized AE reporting frameworks to improve consistency, comparability, and interpretability of safety evidence.

### 4.3. Limitations and Strengths

This study has some limitations. First, this review was restricted to RCTs, which are appropriate for estimating comparative AE risk but may not adequately detect rare, delayed, or operator-related complications. Although the search included the gray literature indexed in the 10 databases, backward citation searching, and examination of registry entries linked to records identified during screening, independent systematic searches of trial registries and safety-surveillance databases were not conducted. Relevant unpublished trials or spontaneous safety reports may therefore have been missed. Accordingly, the present findings should be interpreted as characterizing AEs reported in RCTs, rather than the complete safety profile of TEA. Observational evidence is being evaluated separately in a companion review derived from the same protocol.

Second, methodological limitations in AE assessment and reporting were identified across the included studies. To address these issues, standardized frameworks for AE terminology, causality assessment, and severity grading were applied during data synthesis to harmonize inconsistently reported safety data. Nevertheless, the analysis remained dependent on the quality of the original reports, and inconsistencies in AE definitions, collection methods, and classification may have contributed to residual heterogeneity and reduced the comparability of pooled estimates.

Third, the available evidence was limited by insufficient reporting of key variables, which precluded comprehensive evaluation of potential confounding factors and safety outcomes related to exposure and time, such as dose–response relationships, delayed or persistent AEs, and long-term outcomes.

Fourth, a formal certainty-of-evidence assessment was not performed; therefore, confidence in the pooled estimates should be considered in light of the identified risk of bias, clinical and methodological heterogeneity, and imprecision due to sparse events.

Fifth, the evidence base was geographically concentrated, with most included studies conducted in China. This regional concentration may limit the generalizability of the findings to healthcare settings with different clinical practices, healthcare systems, and AE reporting environments.

Despite these limitations, this study has several important strengths. It synthesized evidence from 90 RCTs across diverse clinical conditions, providing a quantitative overview of TEA safety. By evaluating both overall and intervention-related AEs, the review offered complementary insights into the burden of AEs during treatment and those attributable specifically to TEA. In addition, studies with double-zero events were retained in primary analyses to minimize data loss and maximize available safety data, with sensitivity analyses supporting robustness.

## 5. Conclusions

This study systematically evaluated TEA safety based on 90 RCTs. TEA was primarily associated with mild and transient local AEs, while SAEs were rare. No consistent increase in AE risk was observed across monotherapy or adjunctive intervention settings. However, interpretation should be cautious due to heterogeneity in AE definitions and reporting quality across included studies. Further well-designed trials with standardized AE reporting and longer follow-up are warranted to strengthen the evidence base.

## Figures and Tables

**Table 1:** Characteristics and pooled incidence of thread-embedding-acupuncture-related adverse events.

AE Type	Severity	Treatment and Recovery (No. of Participants)	AE/Participants	Pooled Incidence (95% CI, I^2^)
Pain	Grade 1 (35)	Self-limited within 7 days (33) Resolved after conservative treatment (2)	37/334	6% (0% to 48%, I^2^ = 0%)
	Grade 2 (2)	Persist over 7 days (1) NR (1)		
Hemorrhage	Grade 1 (36)	Self-limited (3) Self-limited within 7 days (26) NR (7)	36/650	4% (2% to 9%, I^2^ = 47.5%)
Nodule /Induration	Grade 1 (38)	Self-limited (12) Self-limited within 2 days (16) Self-limited within 2 weeks (1) Resolved within 1–3 months (3) Resolved after conservative treatment (6)	39/656	4% (1% to 9%, I^2^ = 77.5%)
	UA (1)	NR (1)		
Erythema	Grade 1 (3)	Self-limited within 2 days (1) Self-limited within 1 week (1) NR (1)	10/276	4% (2% to 7%, I^2^ = 0%)
	Grade 2 (2)	Resolved after erythromycin ointment (2)		
	UA (5)	NR (5)		
Swelling	Grade 1 (7)	Self-limited within 2–3 days (3) Self-limited within 1 week (1) Self-limited within several days (2) NR (1)	14/506	3% (2% to 5%, I^2^ = 0%)
	Grade 2 (2)	Resolved after erythromycin ointment (2)		
	UA (5)	NR (5)		
Paresthesia	Grade 1 (4)	Self-limited (3) Resolved after hot pack (1)	4/110	4% (1% to 9%, I^2^ = 0%)
Allergic reaction	Grade 1 (1)	Self-limited after 1 day (1)	3/384	1% (0% to 3%, I^2^ = 0%)
	Grade 2 (2)	Resolved after medication (2)		
Infection	Grade 1 (1)	Self-limited (1)	2/628	0% (0% to 4%, I^2^ = 0%)
	UA (1)	NR (1)		
Pruritus	Grade 1 (1)	NR (1)	1/50	2% (0% to 13%, I^2^ = 0%)
Hypoesthesia	Grade 1 (3)	Self-limited after 3 days (3)	3/50	NA
Irritation	Grade 1 (1)	Resolved after hot pack (1)	1/42	NA
Hyperpigmentation	Grade 1 (1)	Self-limited after 2 months (1)	1/28	NA
Pre-syncope	Grade 2 (2)	Resolved after usual care (1) NR (1)	2/296	1% (0% to 3%, I^2^ = 0%)
Dizziness	UA (2)	NR (2)	2/166	1% (0% to 5%, I^2^ = 0%)
Insomnia	UA (1)	NR (1)	1/106	NA
Nausea	UA (1)	NR (1)	1/60	NA
Other AEs	Grade 1 (2)	NR (2)	2/106	NA

Abbreviations: TEA, Thread-embedding acupuncture; AE, Adverse event; UA, unassessable; NR, not reported; NA, not applicable.

## Data Availability

Data used in the meta-analysis were obtained from the publicly available literature cited in references from [47,48,49,50,51,52,53,54,55,56,57,58,59,60,61,62,63,64,65,66,67,68,69,70,71,72,73,74,75,76,77,78,79,80,81,82,83,84,85,86,87,88,89,90,91,92,93,94,95,96,97,98,99,100,101,102,103,104,105,106,107,108,109,110,111,112,113,114,115,116,117,118,119,120,121,122,123,124,125,126,127,128,129,130,131,132,133,134,135,136].

## Supplementary Materials

The following supporting information can be downloaded at https://www.mdpi.com/article/10.3390/biomedicines14091901/s1, Table S1: PRISMA checklist; Table S2: PRISMA-harms checklist; Table S3: Search strategy; Table S4: Adverse event terminology standardization according to MedDRA; Table S5: Characteristics of included studies; Table S6: Summary of McHarm assessment; Figure S1: Forest plot of the RD of overall AEs in head-to-head comparisons; Figure S2: Forest plot of the RD of overall AEs in add-on therapy comparisons; Figure S3: Forest plot of the RR of intervention-related AEs in head-to-head comparisons; Figure S4: Forest plot of the RD of intervention-related AEs in head-to-head comparisons; Figure S5: Forest plot of the RR and RD of intervention-related AEs in add-on therapy of acupuncture; Figure S6: Forest plot of the RR of overall AEs (excluding double zero event studies); Figure S7: Funnel plots assessing publication bias; Figure S8: Forest plot of the RR and RD of intervention-related AEs in TEA versus sham TEA (sensitivity analysis); Figure S9: Forest plot of the RR of overall AEs in TEA versus CM (sensitivity analysis); Figure S10: Forest plot of the sensitivity analysis of overall AEs in add-on therapy CM; Figure S11: Summary of risk of bias.

## Abbreviations

The following abbreviations are used in this manuscript:

AEs	adverse events
AT	acupuncture treatment
CiNii	Citation Information by NII
CIs	confidence intervals
CM	conventional medication
CNKI	China National Knowledge Infrastructure
CTCAE	Common Terminology Criteria for Adverse Events
HM	herb medicine
ICH	International Council for Harmonisation of Technical Requirements for Pharmaceuticals for Human Use
J-STAGE	Japan Science and Technology Information Aggregator, Electronic
KISS	Korean Studies Information Service System
McHarm	McMaster Tool for Assessing Quality of Harms Assessment and Reporting in Study Reports
MedDRA	Medical Dictionary for Regulatory Activities
OASIS	Open Access Scholarly Information Source
PDO	polydioxanone
PGA	polyglycolic acid
PGLA	polyglycolic acid–lactic acid
PRISMA	Preferred Reporting Items for Systematic Reviews and Meta-Analyses
PT	preferred term
RCTs	randomized controlled trials
RD	risk difference
RoB	risk of bias
RR	risk ratio
SAEs	serious adverse events
SR	systematic review
TEA	thread-embedding acupuncture
WHO-UMC	World Health Organization–Uppsala Monitoring Centre
