# Supplementary material for: Safety Profile of Thread-Embedding Acupuncture: A Systematic Review and Meta-Analysis of Randomized Controlled Trials
Source: Biomedicines. 2026 Aug 26;14(9):1901. doi: 10.3390/biomedicines14091901 (PMC13604257; doi:10.3390/biomedicines14091901)

Table S1 PRISMA Checklist

Section and Topic	Item #	Checklist item	Location where item is reported
**TITLE**			
Title	1	Identify the report as a systematic review.	Title
**ABSTRACT**			
Abstract	2	See the PRISMA 2020 for Abstracts checklist.	Abstract
**INTRODUCTION**			
Rationale	3	Describe the rationale for the review in the context of existing knowledge.	Introduction
Objectives	4	Provide an explicit statement of the objective(s) or question(s) the review addresses.	Last paragraph of introduction
**METHODS**			
Eligibility criteria	5	Specify the inclusion and exclusion criteria for the review and how studies were grouped for the syntheses.	Materials and Methods (2.2 Eligibility Criteria)
Information sources	6	Specify all databases, registers, websites, organisations, reference lists and other sources searched or consulted to identify studies. Specify the date when each source was last searched or consulted.	Materials and Methods (2.1 Search strategy)
Search strategy	7	Present the full search strategies for all databases, registers and websites, including any filters and limits used.	Materials and Methods (2.1 Search strategy)
Selection process	8	Specify the methods used to decide whether a study met the inclusion criteria of the review, including how many reviewers screened each record and each report retrieved, whether they worked independently, and if applicable, details of automation tools used in the process.	Materials and Methods (2.3 Study selection and Data extraction)
Data collection process	9	Specify the methods used to collect data from reports, including how many reviewers collected data from each report, whether they worked independently, any processes for obtaining or confirming data from study investigators, and if applicable, details of automation tools used in the process.	Materials and Methods (2.3 Study selection and Data extraction)
Data items	10a	List and define all outcomes for which data were sought. Specify whether all results that were compatible with	Materials and

Section and Topic	Item #	Checklist item	Location where item is reported
		each outcome domain in each study were sought (e.g. for all measures, time points, analyses), and if not, the methods used to decide which results to collect.	Methods (2.3 Study selection and Data extraction)
	10b	List and define all other variables for which data were sought (e.g. participant and intervention characteristics, funding sources). Describe any assumptions made about any missing or unclear information.	Materials and Methods (2.3 Study selection and Data extraction)
Study risk of bias assessment	11	Specify the methods used to assess risk of bias in the included studies, including details of the tool(s) used, how many reviewers assessed each study and whether they worked independently, and if applicable, details of automation tools used in the process.	Materials and Methods (2.4 Risk of Bias and Harms-reporting quality assessment)
Effect measures	12	Specify for each outcome the effect measure(s) (e.g. risk ratio, mean difference) used in the synthesis or presentation of results.	Materials and Methods (2.8 Statistical Analysis)
Synthesis methods	13a	Describe the processes used to decide which studies were eligible for each synthesis (e.g. tabulating the study intervention characteristics and comparing against the planned groups for each synthesis (item #5)).	Results (first paragraph of 3.2 Risk of TEA-Related AEs, 3.3 Risk of Overall AEs, and 3.4 Risk of Intervention Related AEs)
	13b	Describe any methods required to prepare the data for presentation or synthesis, such as handling of missing summary statistics, or data conversions.	Materials and Methods (2.5 – 2.7sections)
	13c	Describe any methods used to tabulate or visually display results of individual studies and syntheses.	Materials and Methods (last paragraph of 2.8 Statistical Analysis)
	13d	Describe any methods used to synthesize results and provide a rationale for the choice(s). If meta-analysis was performed, describe the model(s), method(s) to identify the presence and extent of statistical heterogeneity, and	Materials and Methods (2.8

Section and Topic	Item #	Checklist item	Location where item is reported
		software package(s) used.	Statistical Analysis)
	13e	Describe any methods used to explore possible causes of heterogeneity among study results (e.g. subgroup analysis, meta-regression).	Materials and Methods (2.8 Statistical Analysis)
	13f	Describe any sensitivity analyses conducted to assess robustness of the synthesized results.	Materials and Methods (2.8 Statistical Analysis)
Reporting bias assessment	14	Describe any methods used to assess risk of bias due to missing results in a synthesis (arising from reporting biases).	Materials and Methods (2.4 Risk of Bias and Harms-reporting quality assessment)
Certainty assessment	15	Describe any methods used to assess certainty (or confidence) in the body of evidence for an outcome.	Materials and Methods (2.8 Statistical Analysis)
**RESULTS**			
Study selection	16a	Describe the results of the search and selection process, from the number of records identified in the search to the number of studies included in the review, ideally using a flow diagram.	Results (Figure 1. PRISMA flow chart)
	16b	Cite studies that might appear to meet the inclusion criteria, but which were excluded, and explain why they were excluded.	Results (3.1 Characteristics of Included studies)
Study characteristics	17	Cite each included study and present its characteristics.	Results (3.1 Characteristics of Included studies)
Risk of bias in studies	18	Present assessments of risk of bias for each included study.	Results (3.8 Quality of Studies)
Results of individual studies	19	For all outcomes, present, for each study: (a) summary statistics for each group (where appropriate) and (b) an effect estimate and its precision (e.g. confidence/credible interval), ideally using structured tables or plots.	Supplementary file S1 (Table S4 Characteristics of Included Studies)

Section and Topic	Item #	Checklist item	Location where item is reported
Results of syntheses	20a	For each synthesis, briefly summarise the characteristics and risk of bias among contributing studies.	Results
	20b	Present results of all statistical syntheses conducted. If meta-analysis was done, present for each the summary estimate and its precision (e.g. confidence/credible interval) and measures of statistical heterogeneity. If comparing groups, describe the direction of the effect.	Results (Figure 2-5), Supplementary file S1 Figure S1-S10)
	20c	Present results of all investigations of possible causes of heterogeneity among study results.	Supplementary file S1 Figure S6-S10)
	20d	Present results of all sensitivity analyses conducted to assess the robustness of the synthesized results.	Supplementary file S1 Figure S6-S10)
Reporting biases	21	Present assessments of risk of bias due to missing results (arising from reporting biases) for each synthesis assessed.	Supplementary file S1 Figure S7)
Certainty of evidence	22	Present assessments of certainty (or confidence) in the body of evidence for each outcome assessed.	Not performed
**DISCUSSION**			
Discussion	23a	Provide a general interpretation of the results in the context of other evidence.	Discussion (4.1 Major findings)
	23b	Discuss any limitations of the evidence included in the review.	Discussion (4.2 Methodological heterogeneity)
	23c	Discuss any limitations of the review processes used.	Discussion (4.3 Limitations and Strength)
	23d	Discuss implications of the results for practice, policy, and future research.	Discussion (Last paragraph of 4.1 Major findings and 4.3 Limitations and Strength)
**OTHER INFORMATION**			
Registration and protocol	24a	Provide registration information for the review, including register name and registration number, or state that the review was not registered.	First paragraph of Material and

Section and Topic	Item #	Checklist item	Location where item is reported
			Methods
	24b	Indicate where the review protocol can be accessed, or state that a protocol was not prepared.	First paragraph of Material and Methods
	24c	Describe and explain any amendments to information provided at registration or in the protocol.	First paragraph of Material and Methods
Support	25	Describe sources of financial or non-financial support for the review, and the role of the funders or sponsors in the review.	Funding
Competing interests	26	Declare any competing interests of review authors.	Conflicts of Interest
Availability of data, code and other materials	27	Report which of the following are publicly available and where they can be found: template data collection forms; data extracted from included studies; data used for all analyses; analytic code; any other materials used in the review.	Data Availability Statement

Table S2 PRISMA-harms Checklist

Section / topic (page no)	Item	PRISMA checklist item	PRISMA harms (minimum)	Recommendations for reporting harms in systematic reviews (desirable)	Check if done
**Title**					
Title (3)	1	Identify the report as a systematic review, meta-analysis, or both.	Specifically mention "harms" or other related terms, or the harm of interest in the review.	—	✔
**Abstract**					
Structured summary (4)	2	Provide a structured summary including, as applicable: background; objectives; data sources; study eligibility criteria, participants, and interventions; study appraisal and synthesis methods; results; limitations; conclusions and implications of key findings; systematic review registration number.	—	Abstracts should report any analysis of harms undertaken in the review, if harms are a primary or secondary outcome.	✔
**Introduction**					
Rationale (5)	3	Describe the rationale for the review in the context of what is already known.	—	It should clearly describe in introduction or in methods section which events are considered harms and provide a clear rationale for the specific harm(s), condition(s), and patient group(s) included in the review.	✔
Objectives (5)	4	Provide an explicit statement of questions being addressed with reference to participants, interventions, comparisons, outcomes, and study design (PICOS).	—	PICOS format should be specified, although in systematic reviews of harms the selection criteria for P, C, and O may be very broad (same intervention may have been used for heterogeneous indications in a diverse range of patients)	✔
**Methods**					
Protocol and registration (6)	5	Indicate if a review protocol exists, if and where it can be accessed (eg, web address), and, if available, provide registration information including registration number.	—	No specific additional information is required for systematic reviews of harms.	✔
Eligibility criteria (6)	6	Specify study characteristics (eg, PICOS, length of follow-up) and report characteristics (eg, years considered, language, publication status) used as criteria for eligibility, giving rationale.	—	Report how handled relevant studies (based on population and intervention) when the outcomes of interest were not reported. Report choices for specific study designs and length of follow-up.	✔
Information sources (7)	7	Describe all information sources (eg, databases with dates of coverage, contact with study authors to identify additional studies) in the search and date last searched.	—	Report if only searched for published data, or also sought data from unpublished sources, from authors, drug manufacturers and regulatory agencies. If includes unpublished data, provide the source and the process of obtaining it.	✔

Search (7)	8	Present full electronic search strategy for at least one database, including any limits used, such that it could be repeated.	—	If additional searches were used specifically to identify adverse events, authors should present the full search process so it can be replicated.	✓
Study selection (8)	9	State the process for selecting studies (ie, screening, eligibility, included in systematic review, and, if applicable, included in the meta-analysis).	—	If only included studies reporting on adverse events of interest, defined if screening was based on adverse event reporting in title/abstract or full text. If no harms reported in the text, report if any attempt was made to retrieve relevant data from authors.	✓
Data collection process (9)	10	Describe method of data extraction from reports (eg, piloted forms, independently, in duplicate) and any processes for obtaining and confirming data from investigators.	—	No specific additional information is required for systematic reviews of harms.	✓
Data items (9)	11	List and define all variables for which data were sought (eg, PICOS, funding sources) and any assumptions and simplifications made.	—	Report the definition of the harm and seriousness used by each included study (if applicable). Report if multiple events occurred in the same individuals, if this information is available. Consider if the harm may be related to factors associated with participants (eg, age, sex, use of medications) or provider (eg, years of practice, level of training). Specify if information was extracted and how it was used in subsequent results. Specify if extracted details regarding the specific methods used to capture harms (active/passive and timing of adverse event).	✓
Risk of bias in individual studies (10)	12	Describe methods used for assessing risk of bias of individual studies (including specification of whether this was done at the study or outcome level), and how this information is to be used in any data synthesis.	—	The risk of bias assessment should be considered separately for outcomes of benefit and harms.	✓
Summary measures (11)	13	State the principal summary measures (eg, risk ratio, difference in means).	—	No specific additional information is required for systematic reviews of harms.	✓
Synthesis of results (11)	14	Describe the methods of handling data and combining results of studies, if done, including measures of consistency (eg, I^2) for each meta-analysis.	Specify how zero events were handled, if relevant.		✓
Risk of bias across studies (11)	15	Specify any assessment of risk of bias that may affect the cumulative evidence (eg, publication bias, selective reporting within studies).	—	Present the extent of missing information (studies without harms outcomes), any factors that may account for their absence, and whether these reasons may be related to the results.	✓

Additional analyses (12)	16	Describe methods of additional analyses (eg, sensitivity or subgroup analyses, meta-regression), if done, indicating which were prespecified.	—	Sensitivity analyses may be affected by different definitions, grading, and attribution of adverse events, as adverse events are typically infrequent or reported using heterogeneous classifications. Report the number of participants and studies included in each subgroup.	✔
**Results**					
Study selection (13)	17	Give numbers of studies screened, assessed for eligibility, and included in the review, with reasons for exclusions at each stage, ideally with a flow diagram.	—	If a review addresses both efficacy and harms, display a flow diagram specific for each (efficacy and harm).	✔
Study characteristics (14)	18	For each study, present characteristics for which data were extracted (eg, study size, PICOS, follow-up period) and provide the citations.	Define each harm addressed, how it was ascertained (eg, patient report, active search), and over what time period.	Add additional characteristics to: "P" (population) patient risk factors that were considered as possibly affecting the risk of the harm outcome. "I" (intervention) professional expertise/skills if relevant (for example if the intervention is a procedure). "T" (time) timing of all harms assessments and the length of follow-up.	✔
Risk of bias within studies (15)	19	Present data on risk of bias of each study and, if available, any outcome level assessment (see item 12).	—	Consider the possible sources of biases that could affect the specific harm under consideration within the review. Sample selection, dropouts and measurement of adverse events should be evaluated separately from the outcomes of benefit as described in item 12, above.	✔
Results of individual studies (16)	20	For all outcomes considered (benefits or harms), present, for each study: (a) simple summary data for each intervention group (b) effect estimates and confidence intervals, ideally with a forest plot.	—	Report the actual numbers of adverse events in each study, separately for each intervention.	✔
Synthesis of results (17)	21	Present results of each meta-analysis done, including confidence intervals and measures of consistency.	Describe any assessment of possible causality.	If included data from unpublished sources, report clearly the data source and the impact of these studies to the final systematic review.	✔
Risk of bias across studies (18)	22	Present results of any assessment of risk of bias across studies (see item 15).	—	No specific additional information is required for systematic reviews of harms. See item 15 above.	✔
Additional analysis (18)	23	Give results of additional analyses, if done (eg, sensitivity or subgroup analyses, meta-regression (see item 16)).	—	No specific additional information is required for systematic reviews of harms.	✔
**Discussion**					

Summary of evidence (18)	24	Summarise the main findings including the strength of evidence for each main outcome; consider their relevance to key groups (eg, healthcare providers, users, and policy makers).	—	No specific additional information is required for systematic reviews of harms.	No
Limitations (18)	25	Discuss limitations at study and outcome level (eg, risk of bias), and at review level (eg, incomplete retrieval of identified research, reporting bias).	—	Recognise possible limitations of meta-analysis for rare adverse events (ie, quality and quantity of data), issues noted previously related to collection and reporting.	✓
Conclusions (19)	26	Provide a general interpretation of the results in the context of other evidence, and implications for future research.	—	State conclusions in coherence with the review findings. When adverse events were not identified we caution against the conclusion that the intervention is “safe,” when, in reality, its safety remains unknown.	✓
**Funding**					
Funding (19)	27	Describe sources of funding for the systematic review and other support (eg, supply of data); role of funders for the systematic review.	—	No specific additional information is required for systematic reviews of harms.	✓

Table S3 Search strategy

Search	Query
# 1	"Catgut"[MeSH]
# 2	((“catgut”[All Fields] OR “thread”[All Fields] OR “threads”[All Fields] OR “needle”[All Fields] OR “acupoint”[All Fields] OR “acupoints”[All Fields] OR “semi permanent”[All Fields]) AND (“embed”[All Fields] OR “embeds”[All Fields] OR “embedded”[All Fields] OR “embedding”[All fields] OR “implantation”[All fields])) OR “embedding therap*”[All Fields]
#3	#1 OR #2
#4	“chemically induced”[SH] OR “adverse effects”[SH] OR “poisoning”[SH] OR “complications”[SH] OR “drug effects”[SH] OR “toxicity”[SH]
#5	“adverse event*”[All Fields] OR “adverse effect*”[All Fields] OR “adverse reaction*”[All Fields] OR “adverse-effects”[All Fields] OR “side effect*”[All fields] OR “side reaction*”[All Fields] OR “side-effect*”[All fields] OR “undesirable event*”[All Fields] OR “undesirable effect*”[All Fields] OR “undesirable reaction*”[All Fields] OR “safe*”[All fields] OR “unsafe”[All fields] OR “intoxication”[All fields] OR “poisoning”[All fields] OR “harm*”[All fields] OR “risk*”[All fields] OR “warning*”[All fields] OR “recall*”[All fields] OR “withdrawn*”[All fields] OR “withdrawal*”[All fields] OR “death*”[All fields] OR “fatal*”[All fields] OR “iatrogenic”[All fields] OR “iatropathogenic”[All Fields]
#6	#4 OR #5
#7	#3 AND #6

Table S4. Adverse event Terminology Standardization According to MedDRA

System Organ Class	Preferred term (PT)	Lowest Level Term	Terms used in studies
General disorders and administration site conditions	Injection site pain	pain	pain, sting, Ache, 疼痛, 胀痛
	Injection/implant site hemorrhage	Bruise	Bruise, Bleeding, Ecchymosis, Subcutaneous congestion, Hematoma, 皮下血肿, 淤青, 皮下出血, 出血, 瘀块
	Injection/implant site nodule	Nodule	Nodule, Erythematous nodule
	Injection/implant site Induration	Induration	Induration, Stiffness, 硬结, 排斥反应
	Injection/implant site infection	Infection	Infection, 感染
	Injection site ulcer	Ulcer	Ulcer
	Injection/implant site hyperpigmentation	Hyperpigmentation	Hyperpigmentation
	Injection/Implant site irritation	Uncomfortable	Irritation, Uncomfortable, Discomfort, 異物感, 穴位埋线局部刺激
	Injection site paranesthesia	Tingling	Tingling
	Asthenia	General weakness	Limb weakness, 疲劳, 乏力
	Injection/implant site swelling	Swelling	Swelling, 肿, 肿胀
	Injection/implant site rash	Rash	皮疹
	Pyrexia	Low grade fever	发热, 低热, 微熱

Immune system disorders	Injection/implant site Urticaria	Urticaria	局部隆起
	Injection site hypersensitivity	Allergic reaction	过敏
Skin and subcutaneous tissue disorders	Pruritus	Pruritus	Pruritus, Itching, 搔痒, 痒
	Injection/implant site erythema	Erythema	Redness, erythema, skin flare, 红, 面部潮红,
Nervous system disorders	Pre-syncope	Pre-syncope	Fainting, 晕针
	Dizziness	Dizziness	Dizziness, Vertigo, 頭暈, 眩暈
	Headache	Headache	Headache, 頭痛
	Injection/implant site paranesthesia	Tingling	Tingling
	Injection/implant site hypoesthesia	Numbness	痲, 麻胀
	Insomnia	Insomnia	Sleepness
	Hypersomnia	Hypersomnia	嗜睡
Gastrointestinal disorder	Gastrointestinal disorder	Gastrointestinal disorder	Loss of appetite, abdominal distension, 胃部不适, 肠胃不适, 胃肠道反应, 消化道反应, 腹泄, 腹胀, 腹痛, 口乾, 口舌干燥, 胃酸, 恶心呕吐, 食欲下降, 厌食
Product issues	Device extrusion	Thread sticking out	Thread sticking out, Thread extrusion, 漏線

Table S5. Characteristics of Included Studies

First Author (year)	Country	Disease	Participants (Treatment/ Control)	Group Comparison	Thread embedding acupuncture						Other Interventions
					Thread Material	Needle Diameter	Treatment site	Interval	Total sessions	Follow-up	
**Al et al, 2020**	China	Bronchiectasis	50/50	TEA+CM+HM vs CM+HM	NR	0.9mm	Thoracic, Spine, Extremity	15days	4sessions	NR	1. CM: Salmeterol/Fluticasone, 50/250 μg, INH twice daily; Erythromycin, 0.25 g, PO twice daily<br>2. HM: Jianpi Qushi Huayu, 20 g, PO, twice daily
**Cai et al, 2018**	China	Allergic rhinitis	40/40	TEA vs CM	absorbable surgical suture	NR	Head, Spine, Extremity	1week	4sessions	NR	1. CM: Levocetirizine 5mg, PO, once daily
**Chen et al, 2010**	China	Postmenopausal osteoporosis	33/32	TEA vs CM	Catgut	0.8mm	Abdomen, Spine, Extremity	2weeks	2sessions	NR	1. CM: Fufuchun Capsules (Ethinylestradiol 0.625 μg, Dydrogesterone 0.25mg, Vitamin A, D, E, Calcium), PO, 2capsule/day
**Chen et al, 2012**	China	Atrial fibrillation	30/30	TEA vs CM	NR	NR	Extremity	1week	2sessions	1year	1. CM: Amiodarone 200mg, three times daily (1 week), twice daily(1week)
**Chen et al, 2014 (a)**	China	Allergic rhinitis	36/33	TEA+AT vs AT	Catgut	0.7mm	Spine	2weeks	2sessions	NR	1. AT: 0.25mmX(25~50)mm, moxibustion 2cm, 30min/session, every other day, 2~3sessions/week
**Chen et al, 2014 (b)**	China	Postherpetic neuralgia	55/56	TEA+AT vs AT	Catgut	NR	Thoracic	10days	9sessions	NR	1. AT: needle NR, 2Hz/100Hz 2~5mA Dense-disperse wave, 30min/session, every other day
**Chen et al, 2018**	China	Obesity	40/40	TEA vs Sham	Catgut	23G	Abdomen, Extremity	1week	4sessions	None	Same protocol with treatment group only without thread
**Chen et al, 2019**	China	Obesity	28/23	TEA vs LM	PGA	0.7mm	Abdomen	2weeks	6sessions	NR	1. LM: Hypocaloric diet 1000-1200kcal/day, 50–60% carbohydrates, 20% protein, < 30% total fat, and 18 g of fiber/1000 kcal; Exercise: 65-85% of maximum heart rate, 30-60min/session, daily
**Chen et al, 2021**	China	Obesity	35/35	TEA (sono-guiding) vs TEA (non-guiding)	PGLA	0.5mm	Abdomen	2weeks	3sessions	NR	None
**Chen et al, 2022**	China	Obesity	106/107	TEA vs Sham	Catgut	0.9mm	Abdomen, Spine, Extremity	2weeks	8sessions	24weeks	Same protocol with treatment group only without thread
**Cheng et al, 2012**	China	Urticaria	56/56	TEA vs CM	Catgut	0.9mm	Abdomen, Spine, Extremity	15days	3sessions	45days	1. CM: Levocetirizine 5mg, PO, once daily / Diphenhydramine 1cc IM inj. every other day
**Cheng et al, 2018**	China	Cervical radiculopathy	73/73	TEA vs AT	absorbable surgical suture	0.8mm	Abdomen	15days	3sessions	NR	1. AT(EA): 0.30x50mm needle, continuous wave, 30min/session, daily

**Choi et al, 2026**	Korea	Overactive bladder	34/32	TEA vs Sham	PDO	30G	Abdomen, Spine	1week	8sessions	8weeks	Same protocol with treatment group only without thread
**Chu et al, 2018**	China	Cervical spondylitic radiculopathy	34/34/34	TEA vs Sham	PGLA	0.7mm	Spine, Extremity	1week	3sessions	7weeks	Group 1 received treatment at a superficial layer, whereas both Group 2 and the sham group received treatment at a deep muscle layer. All other aspects of the protocol were identical across the three groups.
**Dai et al, 2022**	China	Post stroke constipation	42/42	TEA vs Sham	PDO	0.7mm	Abdomen, Spine, Extremity	10days	12sessions	3months	Same protocol with treatment group only without thread
**Deng et al, 2017**	China	Anal fissure	30/30	TEA vs CM	Catgut	0.7mm	Abdomen, Perineum, Extremity	1week	4sessions	3months	1. CM: 0.2% Nitroglycerin ointment, apply TOP, twice daily
**Ding et al, 2015**	China	Chronic facial paralysis	93/93	TEA(Catgut) vs TEA(PDS)	Catgut or PDS	0.7mm	Spine, Extremity	10days	6sessions	NR	The three groups were administered with only different thread materials.
**Ding et al, 2016**	China	Refractory prosopopoeia	76/76	TEA(Catgut) vs TEA(PDS)	Catgut or PDS	0.7mm	Spine, Extremity	10days	6sessions	NR	The three groups were administered with only different thread materials.
**Du et al, 2016**	China	Rheumatoid arthritis	45/42	TEA vs CM	Catgut	0.7mm	Spine, Extremity	2weeks	4sessions	NR	1. CM: MTX 15mg, PO, weekly; Folate 0.4mg, PO once daily; Prednisone 10mg, PO, once daily
**Du et al, 2020**	China	Obesity	105/105	TEA vs Sham	absorbable surgical suture	0.7mm	Abdomen	2weeks	4sessions	1month	Same protocol with treatment group only without thread
**Fu et al, 2018**	China	Allergic rhinitis	36/35	TEA+AT vs AT	PDO	NR	Abdomen, Thoracic	2weeks	2sessions	NR	1. AT: 0.25mmX40mm, 30min/session, every other day
**Ge et al, 2015**	China	Obesity	28/28/28	TEA vs TEA vs TEA	Catgut	0.7mm	Abdomen, Spine, Extremity	1week/ 2weeks/ 4weeks	8sessions/ 4sessions/ 2sessions	NR	The three groups were administered with only different treatment intervals.
**Ge et al, 2019**	China	Vascular Dementia	30/30	TEA vs CM	Catgut	0.35mm	Head, Spine	4weeks	3sessions	NR	1. CM: Donepezil HCl 5mg, PO, once daily
**Gong et al, 2022**	China	Lumbar Sprain	30/30	TEA vs CM	PGLA	0.9mm	Spine, Extremity	1week	2sessions	NR	1. CM: Diclofenac 1% gel (20g), topical, three times daily
**Goo et al, 2022**	Korea	Lumbar herniated intervertebral disc	35/35	TEA vs Sham	PDO	29G	Spine	1week	8sessions	8weeks	Same protocol with treatment group only without thread
**Huang et al, 2020**	China	Chronic low back pain	46/46	TEA+AT vs AT	Catgut	NR	Spine	2weeks	6sessions	NR	1. AT: 0.25mmX25mm, 30min/session, 3sessions/week
**Ji et al, 2021**	China	Stroke	33/33	TEA+AT vs AT	Catgut	0.8mm	Spine	1week	4sessions	NR	1. AT: needle NR, 30min/session, 5sessions/week
**Jia et al, 2018**	China	Osteoporosis	60/60	TEA vs CM	Catgut	NR	Abdomen, Spine, Extremity	10days	9sessions	6months	1. CM: Calcium carbonate 1.5g, PO, once daily; Vitamin D3 125IU, PO, once daily

**Jiang et al, 2020**	China	Polycystic ovarian syndrome	60/60	TEA+ CM +HM vs CM +HM	NR	0.8mm	Abdomen, Spine	2weeks	5sessions	NR	1. CM: Metformin, 500 mg/tablet, PO, twice daily<br>2. HM: CangfuDaotan Decoction, 100 mL, PO, twice daily
**Kim et al, 2021**	Korea	Chronic neck pain	64/64	TEA vs PT	PDO	29G	Spine	1week	4sessions	8weeks	1. PT: Hot pack, 15min/session, Interferential current therapy 15min/session, 2sessions/week
**Kjerkegaard et al, 2011**	Denmark	Cervical osteoarthritis	24/22	TEA vs Sham	Gold	NR	Spine	NA	Single session	NR	Same protocol with treatment group only without thread
**Lee et al, 2018**	Korea	Chronic lumbar back pain	20/20	TEA vs AT	PDO	27G	Abdomen, Spine	1week	6sessions	NR	1. AT(EA): 0.30x40mm needle, 4Hz Continuous wave, 20±5min/session, once daily, 2sessions/week
**Lee et al, 2019**	Korea	Atopic dermatitis	7/7	TEA vs AT	PDO	31G	Extremity	1week	4sessions	NR	1. AT: 0.25X40mm, 20min/session, once daily, 3sessions/week
**Li et al, 2005**	China	Chronic gastritis	36/34	TEA vs AT	Catgut	0.9mm	Abdomne, Spine, Extremity	2weeks	6sessions	NR	1. AT: number 28 needle, 20min/session, once daily, 6sessions/week
**Li et al, 2011**	China	Insomnia	61/61	TEA+AT vs AT	Catgut	0.9mm	Spine	2weeks	2sessions	NR	1. AT: 0.35mmX25mm, 30min/session, 5sessions/week
**Li et al, 2015**	China	Hyperlipidemia	30/30	TEA vs HM	Catgut	0.7mm	Abdomen, Extremity	1week	8sessions	NR	1. HM: Xuezhikang 2Capsule, PO, twice daily
**Li et al, 2016 (a)**	China	Allergic rhinitis	68/68	TEA vs Sham	Catgut	0.9mm	Head, Extremity	2weeks	2sessions	8weeks	Same protocol with treatment group only without thread
**Li et al, 2016 (b)**	China	Diabetic constipation	50/50	TEA+ CM vs TEA vs CM	Catgut	0.9mm	Abdomen, Spine, Extremity	1week	4sessions	NR	1. CM: Forlax 10g, PO, once daily
**Li et al, 2019**	China	Tobacco dependence	50/50	TEA vs CM	absorbable surgical suture	0.7mm	Spine, Extremity	2weeks	4sessions	NR	1. CM: Bupropion 150mg(day 1~3), 300mg(day 4~7), 150mg (after day7), PO, once daily
**Li et al, 2020 (a)**	China	Ulcerative colitis	30/30	TEA vs CM	Catgut	0.2mm	Abdomen, Spine	1week	6sessions	NR	1. CM: Mesalazine 100mg, PO, four times daily
**Li et al, 2020 (b)**	China	Insomnia	34/34	TEA vs AT	PGLA	0.7mm	Head, Spine, Extremity	1week	6sessions	1month	1. AT: 0.30x25mm or 0.30X40mm, 30min/session, every other day, 3sessions/week
**Li et al, 2023**	China	Functional anorectal pain	35/36	TEA vs Sham	PGLA	23G	Abdomen, Spine, Extremity	2weeks	4sessions	NR	Same protocol with treatment group only without thread
**Li et al, 2024**	China	Allergic rhinitis	64/64	TEA vs Sham	PDO	0.7mm	Abdomen, Spine, Extremity	2weeks	2sessions	NR	Same protocol with treatment group only without thread
**Li et al, 2025**	China	Stroke	47/47	TEA+UC vs UC	Catgut	0.7mm	Extremity	1week	4sessions	NR	1. UC: health education, bowel preparation, psychological support, pain management, early mobilization
**Li et al, 2026**	China	Androgenic alopecia	40/40	TEA+ CM vs CM	Catgut	0.7mm	Head, Spine, Extremity	1month	6sessions	NR	1. CM: Finasteride 1mg, PO, once daily

**Lin et al, 2013**	China	Chronic atrophic gastritis	68/62	TEA+HM vs HM	Catgut	NR	Abdomen, Spine, Extremity	4weeks	3sessions	NR	1. HM: Chinese herbal medicine, 1 decoction/day, PO, twice daily
**Liu et al, 2023**	China	Chronic persistent bronchial asthma	45/45	TEA+ CM vs CM	Catgut	1.2mm	Thoracic, Spine, Extremity	15days	4sessions	NR	1. CM: budesonide/formoterol powder Inhaler, 160~320μg, INH, twice daily
**Liu et al, 2024**	China	Obesity	41/41	TEA+AT+UC vs AT+UC	PGLA	NR	Abdomen, Spine, Extremity	15days	4sessions	NR	1. UC: including, diet control, physical activity, no more details reported<br>2. AT: No details of protocol
**Liu et al, 2025**	China	Diminished ovarian reserve	35/35	TEA vs AT	Catgut	0.7mm	Abdomne, Spine, Extremity	2weeks	6sessions	NR	1. AT: Needle NR, 30min/session, 3sessions/week
**Lu et al, 2009**	China	Postherpetic neuralgia	32/28	TEA vs Sham	Catgut	18G	NR	NA	Single session	NR	Same protocol with treatment group only without thread
**Lu et al, 2021**	China	Trigeminal neuralgia	100/100	TEA+AT vs AT	Catgut	1.2mm	Head	10days	3sessions	6months	1. AT: 0.35mmX50mm, 10-30min/session, 7sessions/week
**Luo et al, 2016**	China	Bell's palsy	30/30	TEA+AT vs AT	Catgut	0.9mm	Head, Extremity	15-30days	NR	NR	1. AT(EA): needle NR, EA protocol NR, 7sessions/week
**Ma et al, 2016**	China	Insomnia	25/26	TEA vs CM	Catgut	NR	Spine, Extremity	10days	3sessions	NR	1. CM: Estazolam, once daily 30minuites before sleeping
**Nguyen et al, 2023**	Vietnam	Seizure	27/27	TEA vs Sham	PDO	31G	Head, Spine, Extremity	4weeks	3sessions	4weeks	Same protocol with treatment group only without thread
**Niu et al, 2015**	China	Functional dyspepsia	38/37	TEA+ CM vs CM	Catgut	NR	Abdomen, Extremity	10days	3sessions	NR	1. CM: Trimebutine maleate and Domperidone, PO, three times daily
**Niu et al, 2022**	China	Allergic rhinitis	40/33	TEA+AT vs AT	PGLA	0.7mm	Spine	2weeks	2sessions	6months	1. AT: 0.25mmX40mm or 0.25X25mm, 20min/session, every other day
**Park et al, 2026**	Korea	Chronic rotator cuff disease	32/32	TEA vs Sham	PDO	29G	Extremity	1week	8sessions	8weeks	Same protocol with treatment group only without thread
**Peng et al, 2025**	China	Obesity	31/30	TEA vs AT	Catgut	0.9mm	Abdomen, Spine, Extremity	1week	8sessions	NR	1. AT: 0.30X50mm, 30min/session, once daily, 5sessions/week
**Qiu et al, 2026**	China	Diabetic peripheral neuropathy	49/49	TEA+ CM vs CM	absorbable surgical suture	0.8mm	Abdomen, Spine, Extremity	1week	4sessions	3months	1. CM: Methylcobalamin 5mg, PO, three times daily
**Ren et al, 2022**	China	Obesity	30/30/30/30	TEA vs TEA vs TEA vs TEA	PGA	NR	Abdomen, Spine, Extremity	1week/ 2weeks/ 3weeks/ 4weeks	12sessions/ 6sessions/ 4sessions/ 3sessions	NR	The four groups were administered with only different treatment intervals.
**Shi et al, 2021**	China	Migraine	40/40	TEA vs AT	PGLA	0.7mm	Spine	1week	3sessions	NR	1. AT: Needle NR, 30min/session, once daily, 7sessions/week
**Su et al, 2022**	China	Knee osteoarthritis	43/43	TEA vs AT	absorbable surgical suture	0.7mm	Extremity	1week	4sessions	NR	1. AT: 0.25×40mm, 20min/session, every other day, 3seesions/week

**Sun et al, 2018**	China	Cervical spondylotic radiculopathy	30/30	TEA (sono guiding) vs TEA (non-guiding)	PGLA	0.9mm	Spine	1week	3sessions	NR	None
**Sung et al, 2020**	Korea	Chronic lumbar back pain	19/19	TEA+AT vs AT	PDO	29G	Spine	1week	8sessions	12weeks	1. AT: 0.25x40mm, 20min/session, 2sessions/week
**Tao et al, 2024**	China	Migraine	31/33	TEA vs CM vs TEA+ CM	Catgut	0.7mm	Spine	2weeks	4sessions	4weeks	1. CM: Metoprolol tartrate 12.5mg, PO, two times daily
**Wan et al, 2022**	China	Obesity	72/72	TEA vs Sham	PGLA	0.6mm	Abdomen, Spine	2weeks	6sessions	3months	Same protocol with treatment group only without thread
**Wang et al, 2020**	China	Hyperlipidemia	40/40	TEA(PGA) vs TEA(Catgut)	PGA or Catgut	0.7mm	Head, Abdomen, Spine, Extremity	NR	NR	NR	None
**Wang et al, 2021**	China	Chronic atrophic gastritis	30/30	TEA+ CM +HM vs CM +HM	PDO	NR	Abdomen, Spine, Extremity	10days	3sessions	NR	1. CM: Furazolidone, 0.1 g, PO three times daily–four times daily; Amoxicillin, 0.5 g, PO three times daily–four times daily; Colloidal bismuth pectin, 0.1–0.15 g, PO four times daily; Omeprazole, 20 mg, PO twice daily<br>2. HM: Weifuchun, 1.44 g, PO three times daily
**Wu et al, 2020**	China	Polycystic ovarian syndrome	31/31	TEA vs CM	absorbable surgical suture	NR	Abdomen, Spine, Extremity	10days	9sessions	NR	1. CM: Metformin 500 mg, PO, three times daily; Diane-35, PO, once daily (from day 5 of the menstrual cycle for 21 days per cycle, for three cycles)
**Wu et al, 2022**	China	Chronic nonspecific low back pain	46/46	TEA+AT vs AT	Catgut	NR	Spine	2weeks	4sessions	NR	1. AT: 0.25X25mm, 30min/session, every other day, 3sessions/week
**Xu et al, 2019**	China	Insomnia	170/170/170	TEA vs AT vs CM	Catgut	0.7mm	Abdomen, Spine, Extremity	10days	3sessions	NR	1. AT: 0.25×40mm, 20min/session, once daily, 5seesions/week<br>2. CM: Estazolam 1mg, PO, once daily
**Yang et al, 2020**	China	Obesity	31/32	TEA vs TEA	NR	NR	Abdomen, Spine, Extremity	1week/ 2weeks	12sessions/ 6sessions	NR	The two groups were administered with only different treatment intervals.
**Yang et al, 2024**	China	Pelvic pain	40/40	TEA+HM vs HM	Catgut	NR	Abdomen, Extremity	10days	9sessions	NR	1. HM: Si Ni Si Miao Shi Xiao San, PO, three times daily
**Yang et al, 2025 (a)**	China	Obesity	60/60	TEA vs Sham	Catgut	NR	Abdomen, Extremity	2weeks	6sessions	12weeks	Same protocol with treatment group only without thread
**Yang et al, 2025 (b)**	China	Urticaria	54/54	TEA+ CM vs CM	Catgut	NR	Spine, Extremity	2weeks	2sessions	NR	1. CM: Loratadine 10mg, PO, once daily
**Yao et al, 2017**	China	Obesity	20/20/20	TEA vs TEA vs TEA	Catgut	0.7mm	Abdomen, Spine, Extremity	1week/ 2weeks/ 3weeks	6sessions/ 3sessions/ 2sessions	NR	The three groups were administered with only different treatment intervals.
**Yin et al, 2022**	China	Constipation	59/58	TEA vs CM	Catgut	0.7mm	Abdomen, Spine, Extremity	2weeks	2sessions	3months	1. CM: Lactulose 15ml, twice daily

**Zhang et al, 2003**	China	Psoriasis	88/72	TEA+HM vs HM	Catgut	1.2mm	Abdomen, Spine, Extremity	10days	3sessions	NR	1. HM: Qingdai pill, details NR
**Zhang et al, 2012**	China	Constipation	119/117	TEA vs AT	Catgut	0.7mm	Abdomen	15days	4sessions	NR	1. AT: 0.25x40mm, 30min/session, once every 3days
**Zhang et al, 2021**	China	Hyperlipidemia	39/39	TEA vs HM	Catgut	NR	Abdomen, Spine, Extremity	1week	8sessions	NR	1. HM: Xuezhikang 2Capsule, PO, twice daily
**Zhang et al, 2023**	China	Obesity	69/68	TEA (acupoint) vs TEA (non-acupoint)	Catgut	0.8mm	Abdomen, Spine, Extremity	2weeks	6sessions	4weeks	The two groups were administered with only different treatment points
**Zhang et al, 2025 (a)**	China	Obesity	48/48	TEA vs Sham	PDO	0.9mm	Abdomen, Spine	15days	4sessions	NR	Same protocol with treatment group only without thread
**Zhang et al, 2025 (b)**	China	Allergic rhinitis	52/52/52	TEA vs HM vs TEA+HM	Catgut	NR	Head, Spine, Extremity	1week	4sessions	NR	1. HM: Modified Yiqi Quxie Cang'erzi decoction (granules), twice daily
**Zhang et al, 2025 (c)**	China	Chronic atrophic gastritis	40/40/40/40	TEA vs HM vs TEA+HM vs CM	Catgut	0.7mm	Abdomen, Spine, Extremity	2weeks	12sessions	NR	1. CM: Teprenone 50mg, PO, three time daily<br>2. HM: Xiangsha Wenzhong decoction, 200 mL, twice daily
**Zhao et al, 2019**	China	Spondylosis	45/45	TEA vs AT	PGLA	0.9mm	Spine	2weeks	2sessions	NR	1. AT: 30min/session, daily
**Zhao et al, 2025**	China	Constipation	30/30	TEA vs AT	NR	0.9mm	Abdomen, Spine	1week	12sessions	NR	1. AT: Needle NR, 30min/session, daily
**Zheng et al, 2024**	China	Stroke	43/43	TEA+RH vs RH	Catgut	0.7mm	Extremity	2weeks	2sessions	NR	1. RH: (limb positioning, assisted upper-limb elevation, scapular exercises, weight-bearing, rolling training, and hand function training), 2~4sessions/day
**Zhou et al, 2015**	China	Tinnitus	33/36	TEA+AT vs AT	Catgut	NR	Spine	2weeks	4sessions	4weeks	1. AT: 0.35mmX25mm, 50Hz continuous wave, 30min/session, 5sessions/week
**Zhou et al, 2023**	China	Type 2 diabetic mellitus	30/30	TEA+ CM +LM vs CM +LM	Catgut	0.7mm	Abdomen, Spine, Extremity	1week	8sessions	NR	1. LM: Aerobic exercise, moderate intensity, at least 150min; dietary intervention<br>2. CM: Metformin 1g, PO, twice daily
**Zhu et al, 2024**	China	Obesity	29/28	TEA+LM vs LM	PGA	0.8mm	Abdomen, Spine	10days	6sessions	NR	1. LM: Aerobic exercise, moderate intensity, 40-60min/session, 2-5sessions/week; dietary intervention

Abbreviations: TEA, Thread embedding acupuncture; CM, Conventional medicine; HM, Herb medicine; NR, Not reported; PO, Per os; INH, Inhalation; AT, Acupuncture; IM, Intramuscular; EA, Electroacupuncture; PGA, Polyglycolic acid; PGLA, poly(glycolide-co-lactide); PDO, polydioxanone; LM, Life Modification; TOP, Topical; Hz, Hertz; mA, Milliampere; MTX, Methotrexate; PT, Physical therapy; NA, not applicable; UC, Usual care; RH, Rehabilitation therapy

Table S6. Summary of McHarm Assessment

	McHarm														
**ID**	**Q1**	**Q2**	**Q3**	**Q4**	**Q5**	**Q6**	**Q7**	**Q8**	**Q9**	**Q10**	**Q11**	**Q12**	**Q13**	**Q14**	**Q15**
**Al et al, 2020**	N	N	N	N	N	N	N	N	N	N	N	N	Y	Y	Y
**Cai et al, 2018**	N	N	N	N	N	N	Y	N	N	N	N	N	Y	Y	Y
**Chen et al, 2010**	N	N	N	N	N	N	N	N	N	N	N	Y	Y	Y	Y
**Chen et al, 2012**	N	N	N	Y	N	N	N	N	N	N	N	N	Y	Y	Y
**Chen et al, 2014 (a)**	N	N	N	Y	N	N	N	N	N	N	N	Y	N	Y	N
**Chen et al, 2014 (b)**	N	N	N	N	N	N	N	N	N	N	N	Y	N	Y	N
**Chen et al, 2018**	N	N	N	Y	N	N	N	N	N	N	N	Y	N	Y	Y
**Chen et al, 2019**	N	N	N	Y	Y	N	N	N	N	N	Y	Y	Y	Y	N
**Chen et al, 2021**	Y	N	N	N	N	N	N	N	N	N	Y	Y	Y	Y	Y
**Chen et al, 2022**	N	N	N	Y	N	N	Y	Y	N	N	Y	Y	Y	Y	Y
**Cheng et al, 2012**	N	N	N	N	N	N	N	N	N	N	N	N	Y	Y	N
**Cheng et al, 2018**	N	N	N	N	N	N	N	N	N	N	N	N	Y	U	U
**Choi et al, 2026**	Y	N	N	Y	Y	N	Y	N	N	Y	Y	Y	Y	Y	N
**Chu et al, 2018**	N	Y	N	Y	N	Y	Y	N	N	N	Y	Y	Y	Y	N
**Dai et al, 2022**	N	N	N	Y	N	N	N	N	N	N	N	Y	Y	Y	N
**Deng et al, 2017**	N	N	N	Y	N	N	Y	N	N	N	N	Y	Y	Y	N
**Ding et al, 2015**	N	N	N	N	N	N	N	N	Y	N	Y	N	N	N	Y
**Ding et al, 2016**	N	N	N	N	N	N	N	N	Y	N	Y	N	N	N	Y
**Du et al, 2016**	N	N	N	N	N	N	N	N	N	N	N	Y	Y	Y	Y
**Du et al, 2020**	N	N	N	U	Y	N	N	N	Y	N	Y	Y	Y	Y	U
**Fu et al, 2018**	N	N	N	N	N	N	N	N	N	N	N	N	Y	Y	Y
**Ge et al, 2015**	N	N	N	N	N	N	N	N	N	N	N	N	Y	Y	Y
**Ge et al, 2019**	N	N	N	N	N	N	N	N	N	N	N	N	Y	N	Y
**Goo et al, 2022**	N	N	N	Y	Y	Y	Y	Y	Y	U	Y	Y	N	Y	N
**Gong et al, 2022**	N	N	N	N	N	N	N	N	N	N	N	N	Y	Y	Y
**Huang et al, 2020**	N	N	N	N	N	N	N	N	N	N	N	Y	Y	Y	Y
**Ji et al, 2021**	N	N	N	N	N	N	N	N	N	N	N	Y	Y	Y	N

**Jia et al, 2018**	N	N	Y	N	N	N	N	N	N	N	N	Y	Y	Y	Y
**Jiang et al, 2020**	N	N	N	N	N	N	N	N	N	N	N	Y	Y	Y	Y
**Kim et al, 2021**	Y	N	Y	Y	Y	N	Y	Y	Y	N	Y	Y	N	Y	N
**Kjerkegaard et al, 2011**	N	N	N	Y	N	N	N	N	N	N	N	N	Y	U	U
**Lee et al, 2018**	N	N	N	Y	Y	Y	Y	Y	Y	N	Y	Y	Y	Y	Y
**Lee et al, 2019**	N	N	N	Y	Y	N	N	N	Y	N	Y	Y	Y	Y	U
**Li et al, 2005**	Y	N	N	N	N	N	N	N	N	N	Y	N	Y	Y	N
**Li et al, 2011**	N	N	N	N	N	N	N	N	N	N	N	Y	Y	Y	N
**Li et al, 2015**	N	N	N	N	N	N	N	N	N	N	N	N	Y	Y	N
**Li et al, 2016 (a)**	N	N	N	Y	N	Y	N	Y	N	U	Y	Y	N	Y	N
**Li et al, 2016 (b)**	N	N	N	N	N	N	N	N	Y	N	N	N	Y	Y	Y
**Li et al, 2019**	N	N	N	Y	Y	N	Y	N	Y	N	Y	Y	Y	Y	Y
**Li et al, 2020 (a)**	N	N	N	N	N	N	N	N	N	N	N	N	Y	Y	N
**Li et al, 2020 (b)**	N	N	N	N	N	N	N	N	Y	N	N	N	Y	Y	Y
**Li et al, 2023**	N	N	N	Y	N	N	Y	N	Y	N	Y	Y	Y	Y	U
**Li et al, 2024**	Y	N	N	Y	N	N	N	N	N	N	Y	N	N	Y	Y
**Li et al, 2025**	N	N	N	N	N	N	Y	N	N	N	N	N	N	Y	Y
**Li et al, 2026**	N	N	N	N	N	N	N	N	N	N	N	N	Y	Y	Y
**Lin et al, 2013**	N	N	N	N	N	N	N	N	N	N	N	N	Y	Y	U
**Liu et al, 2023**	N	N	N	N	N	N	N	N	N	N	N	N	Y	Y	Y
**Liu et al, 2024**	N	N	N	N	N	N	N	N	N	N	N	N	Y	Y	Y
**Liu et al, 2025**	N	N	Y	Y	N	N	N	N	Y	Y	Y	Y	Y	Y	Y
**Lu et al, 2009**	N	N	N	N	N	N	N	N	N	N	N	Y	Y	Y	Y
**Lu et al, 2021**	N	N	N	N	N	N	N	N	N	N	N	N	Y	Y	Y
**Luo et al, 2016**	N	N	N	Y	N	N	N	N	N	N	N	N	Y	Y	N
**Ma et al, 2016**	N	N	N	N	N	N	N	N	N	N	N	Y	Y	Y	N
**Nguyen et al, 2023**	N	N	N	Y	Y	Y	N	N	Y	N	Y	Y	Y	Y	N
**Niu et al, 2015**	N	N	N	N	N	N	N	N	N	N	N	N	Y	U	U
**Niu et al, 2022**	N	N	N	N	N	N	N	N	N	N	N	N	Y	Y	Y

**Park et al, 2026**	Y	N	Y	N	N	Y	Y	Y	Y	U	N	Y	Y	Y	N
**Peng et al, 2025**	N	N	N	N	N	N	N	N	N	N	N	N	Y	Y	Y
**Qiu et al, 2026**	N	N	N	N	N	N	N	N	N	N	N	Y	Y	Y	N
**Ren et al, 2022**	N	N	N	N	N	N	N	N	N	N	N	Y	Y	Y	Y
**Shi et al, 2021**	N	N	N	N	N	N	N	N	N	N	N	N	Y	Y	U
**Su et al, 2022**	N	N	N	N	N	N	N	N	N	N	N	N	Y	Y	Y
**Sun et al, 2018**	N	N	N	N	N	N	N	N	N	N	Y	N	Y	Y	N
**Sung et al, 2020**	N	N	N	Y	N	N	Y	Y	N	N	Y	Y	Y	Y	Y
**Tao et al, 2024**	N	N	N	Y	N	N	N	N	Y	N	Y	Y	N	Y	N
**Wan et al, 2022**	N	N	N	Y	N	N	N	N	Y	N	Y	Y	N	N	Y
**Wang et al, 2020**	N	N	N	N	N	N	N	N	N	N	N	N	Y	Y	N
**Wang et al, 2021**	N	N	N	N	N	N	N	N	N	N	N	N	Y	Y	Y
**Wu et al, 2020**	N	N	N	N	N	N	N	N	N	N	N	Y	Y	Y	Y
**Wu et al, 2022**	N	N	N	N	N	N	N	N	N	N	Y	N	Y	Y	Y
**Xu et al, 2019**	N	N	N	N	N	N	N	N	N	N	N	Y	Y	N	N
**Yang et al, 2020**	N	N	N	N	N	N	N	N	N	N	N	Y	Y	Y	Y
**Yang et al, 2024**	Y	N	N	Y	N	N	N	N	N	N	N	Y	N	Y	N
**Yang et al, 2025 (a)**	N	N	N	Y	N	N	Y	N	N	N	N	Y	N	Y	Y
**Yang et al, 2025 (b)**	N	N	N	N	N	N	N	N	N	N	N	N	Y	Y	Y
**Yao et al, 2017**	N	N	N	N	N	N	N	N	N	N	N	N	Y	Y	Y
**Yin et al, 2022**	N	N	N	Y	N	N	N	N	N	N	Y	Y	Y	Y	Y
**Zhang et al, 2003**	N	N	N	N	N	N	N	N	N	N	N	N	N	Y	N
**Zhang et al, 2012**	N	N	N	Y	N	N	N	N	N	N	Y	Y	Y	Y	U
**Zhang et al, 2021**	N	N	N	N	N	N	N	N	N	N	N	N	Y	Y	Y
**Zhang et al, 2023**	N	N	N	Y	N	N	N	N	Y	N	Y	Y	Y	Y	Y
**Zhang et al, 2025 (a)**	N	N	N	Y	N	N	N	N	N	N	N	Y	Y	Y	Y
**Zhang et al, 2025 (b)**	N	N	N	N	N	N	N	N	N	N	N	N	Y	Y	Y
**Zhang et al, 2025 (c)**	N	N	N	N	N	N	N	N	N	N	N	Y	Y	Y	Y
**Zhao et al, 2019**	N	N	N	N	N	N	N	N	N	N	N	N	Y	U	Y
**Zhao et al, 2025**	N	N	N	N	N	N	N	N	N	N	N	N	Y	Y	Y
**Zheng et al, 2024**	N	N	N	N	N	N	N	N	N	N	N	Y	Y	Y	Y

**Zhou et al, 2015**	N	N	N	N	N	N	Y	N	N	N	N	Y	Y	Y	N
**Zhou et al, 2023**	N	N	Y	N	N	N	N	N	N	N	Y	N	Y	Y	Y
**Zhu et al, 2024**	N	N	N	Y	N	N	Y	Y	N	N	N	Y	Y	N	N

Abbreviations: Y, Yes; N, No; U, Unclear

Figure S1. Forest Plot of the RD of Overall AEs in head-to-head Comparisons [55–97].

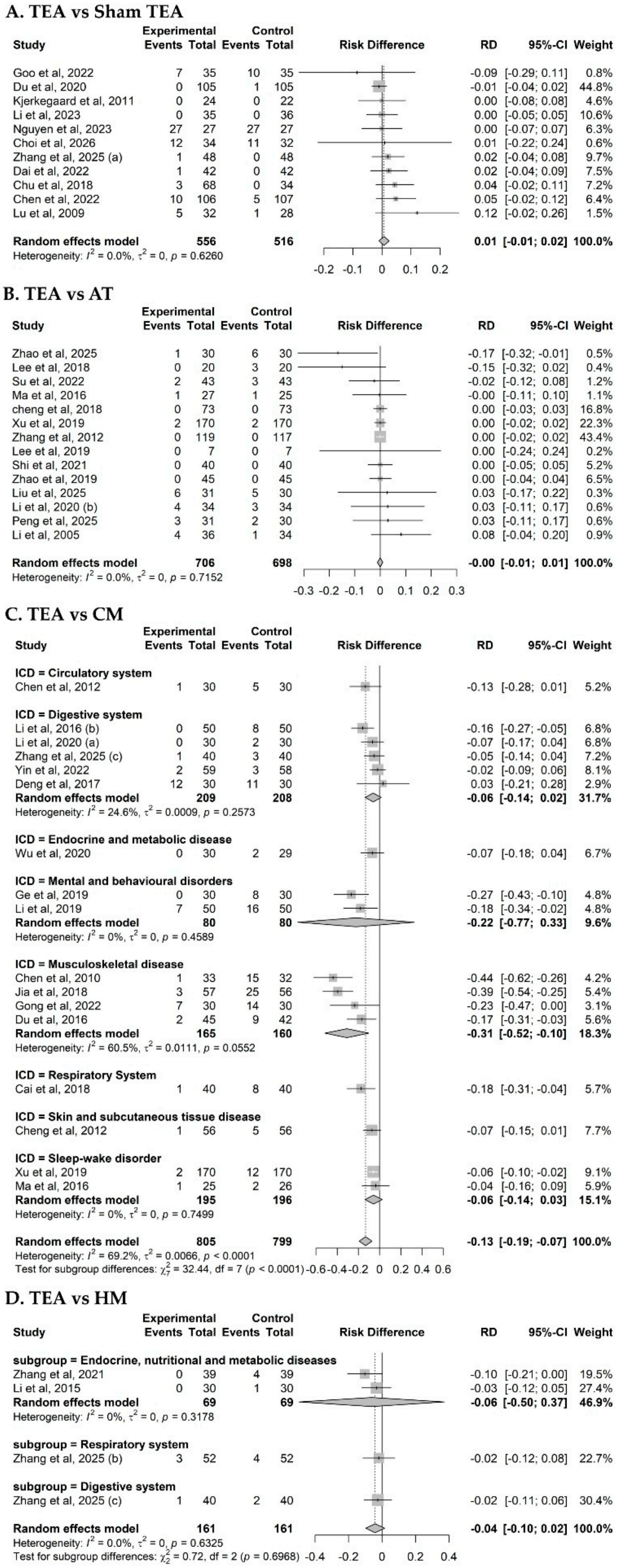

Figure S2. Forest Plot of the RD of Overall AEs in Add-on Therapy Comparisons [55–117].

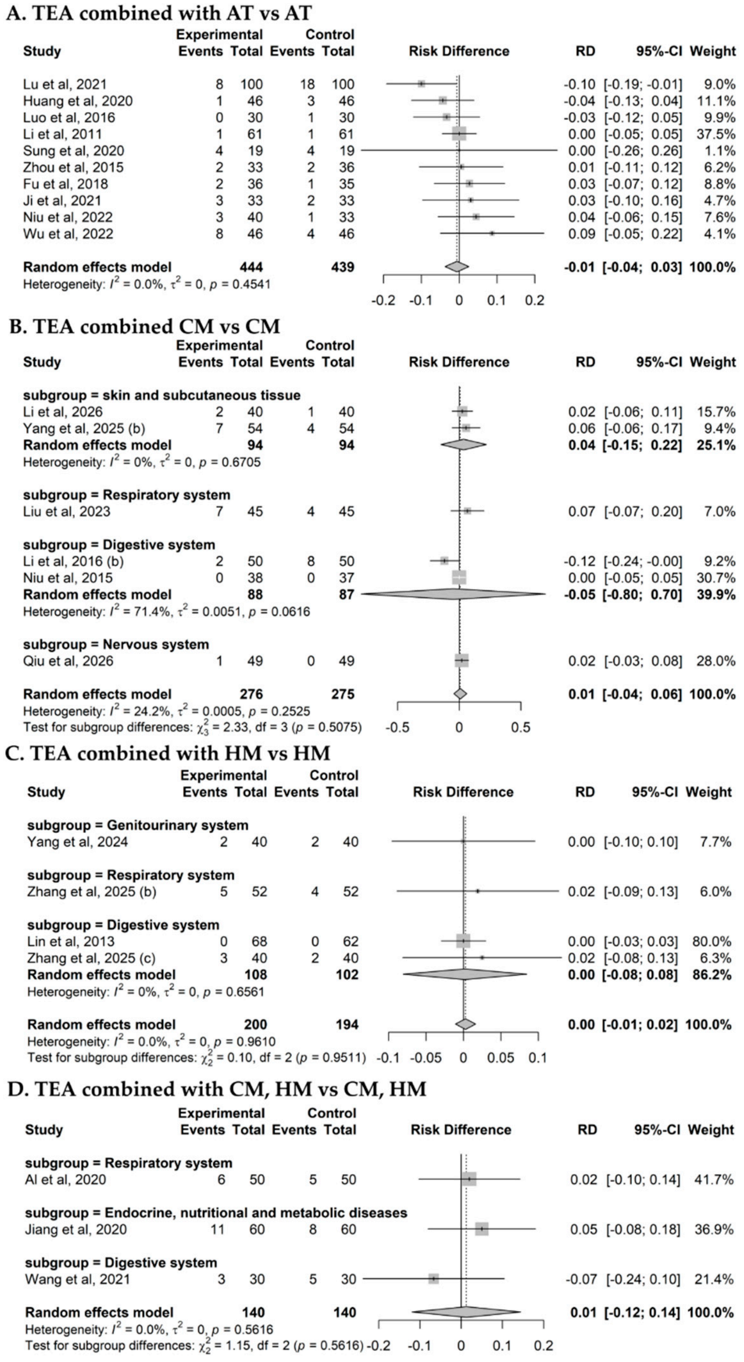

Figure S3. Forest Plot of the RR of Intervention Related AEs in Head-to-Head Comparisons [55–71,88].

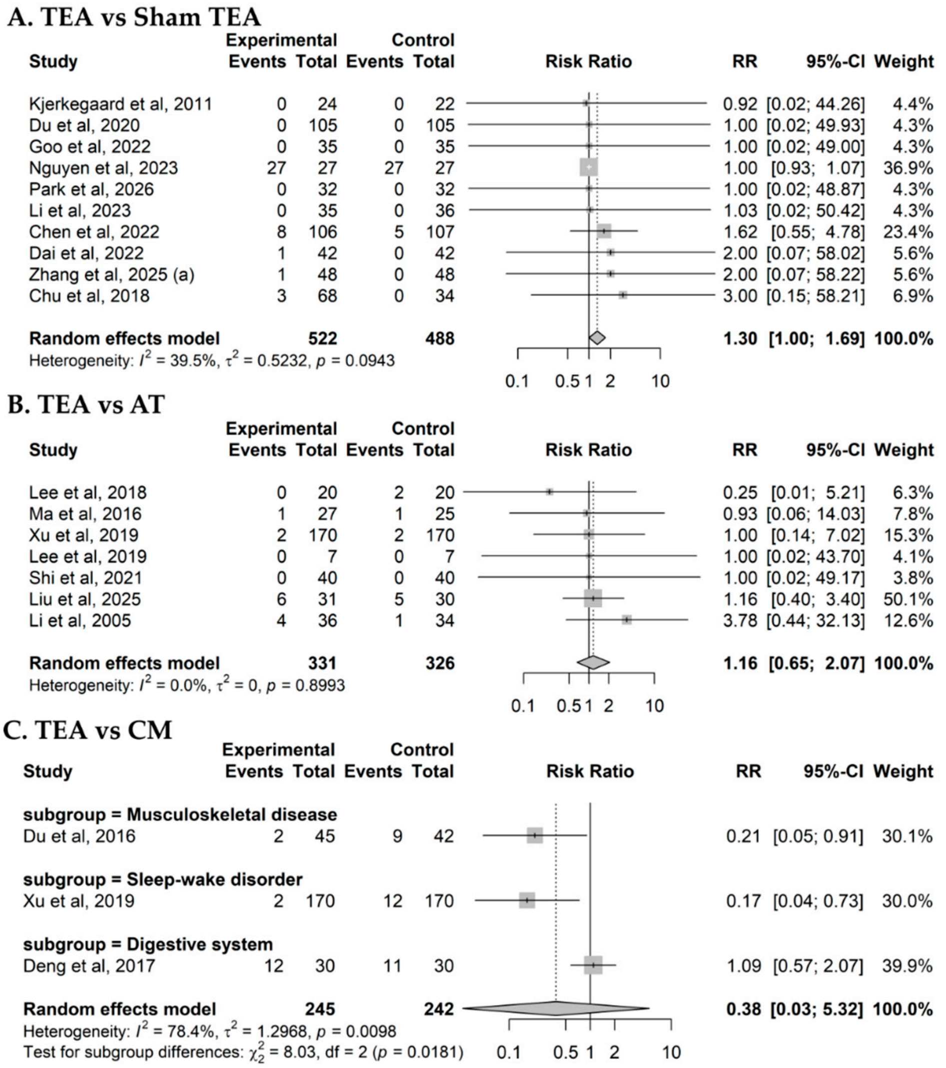

Figure S4. Forest Plot of the RD of Intervention Related AEs in Head-to-Head Comparisons [55–71,88].

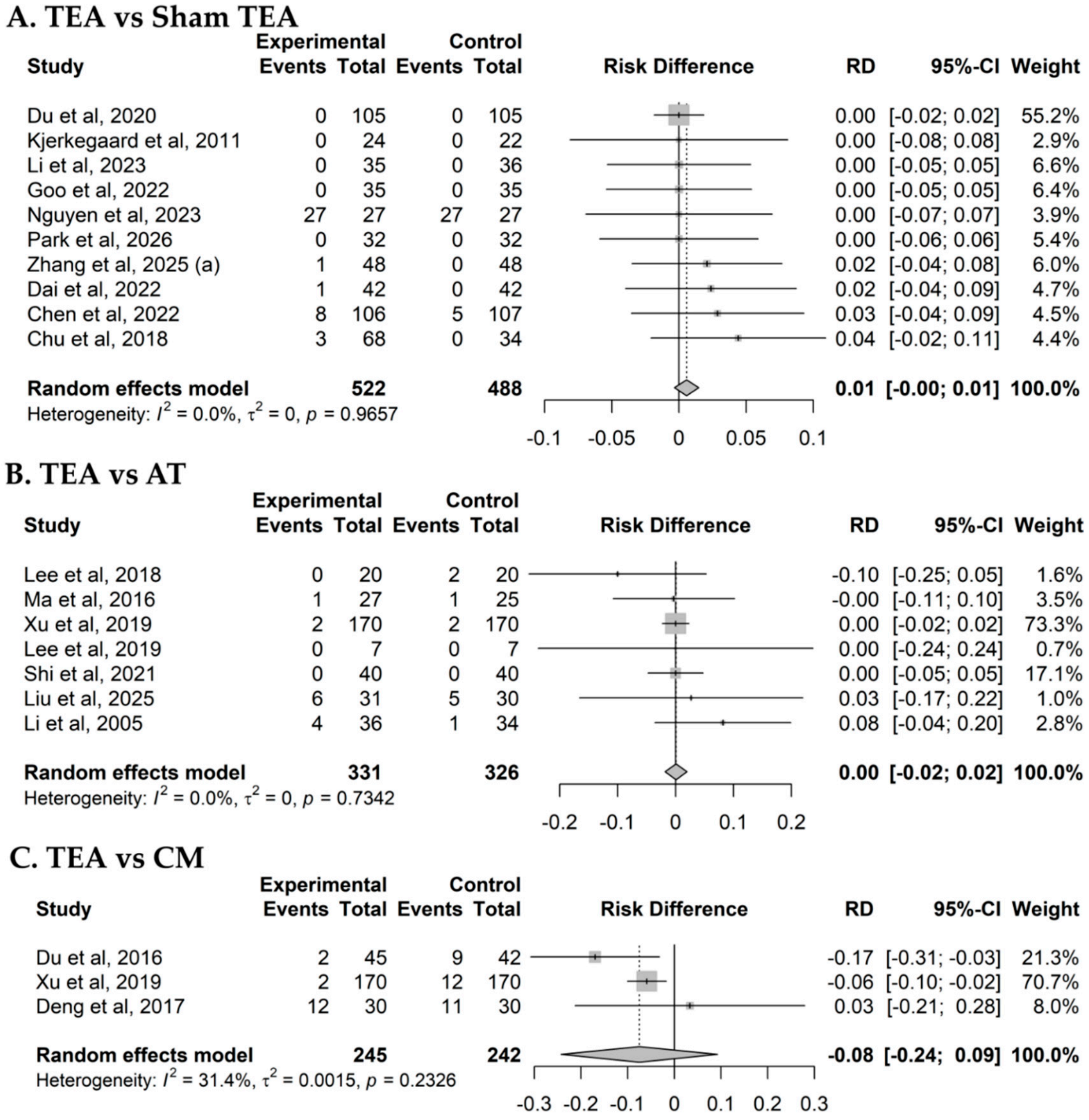

Figure S5. Forest Plot of the RR and RD of Intervention Related AEs in Add-on Therapy of Acupuncture [98,100,102–104,107].

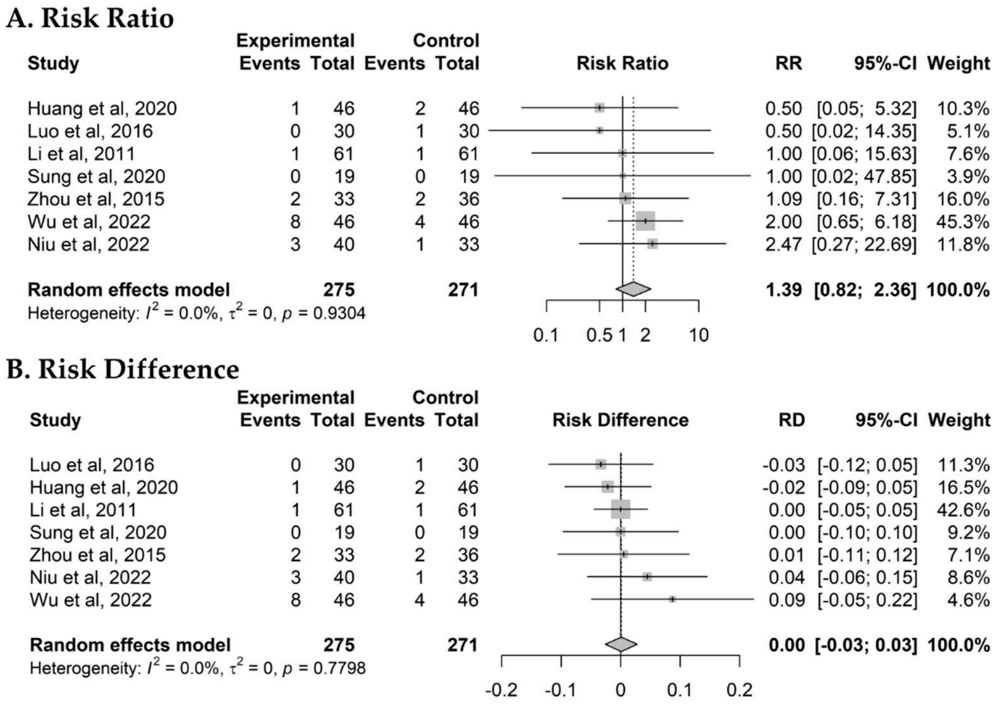

Figure S6. Forest Plot of the RR of Overall AEs (Excluding Double Zero Event Studies) [55–57,59,61,64,65,67–72,84,86,90,91,93,95–97,99,101,108,109,114]

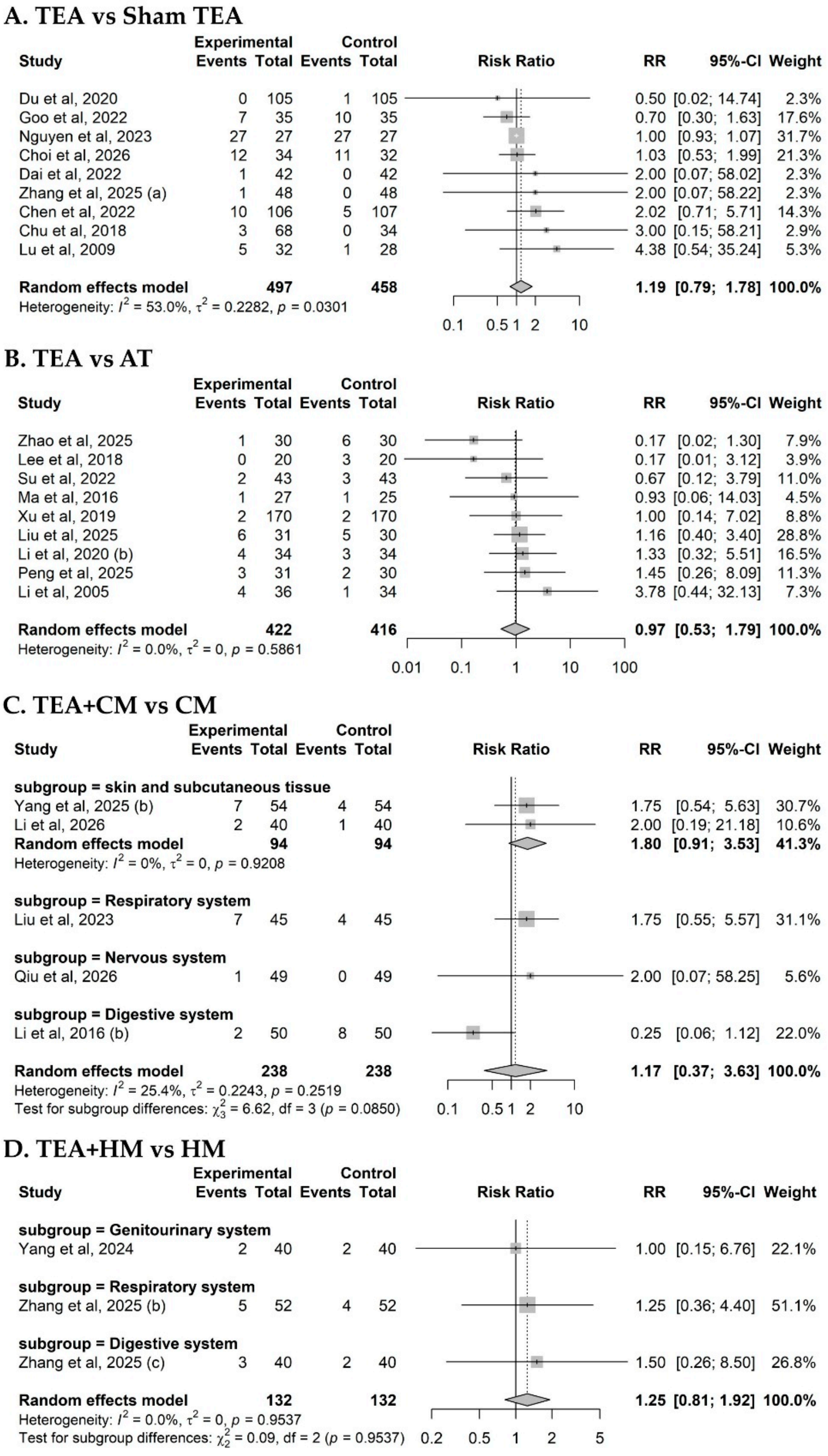

Figure S7. Funnel Plots Assessing Publication Bias

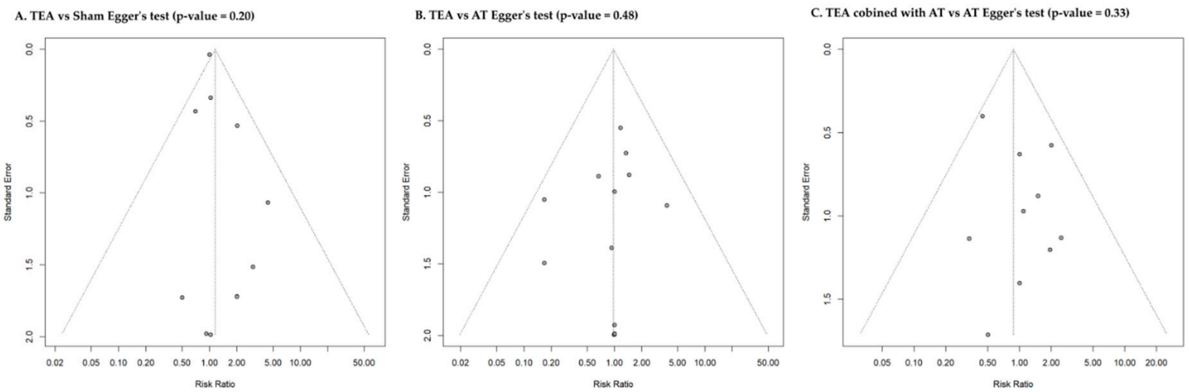

Figure S8. Forest Plot of the RR and RD of Intervention Related AEs in TEA versus Sham TEA (Sensitivity Analysis) [54,56,57,59,61,62,66,68,71].

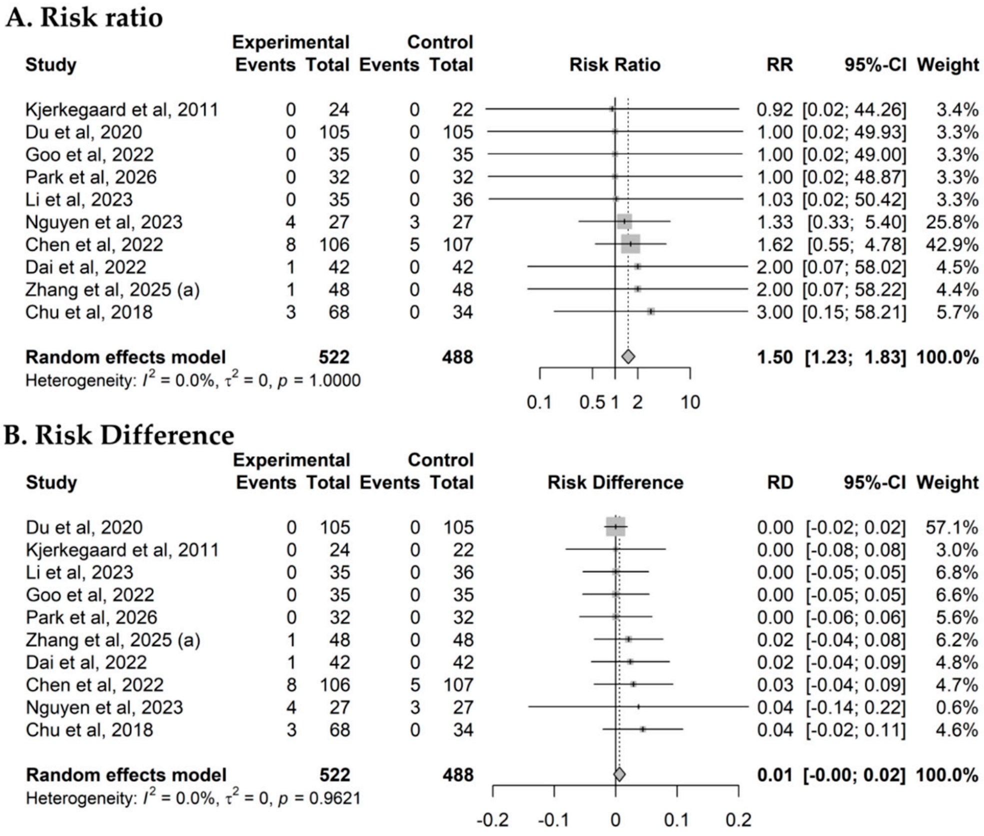

Figure S9. Forest Plot of the RR of Overall AEs in TEA versus CM (Sensitivity Analysis) [60,69,70,74–82,85,92,96,97]

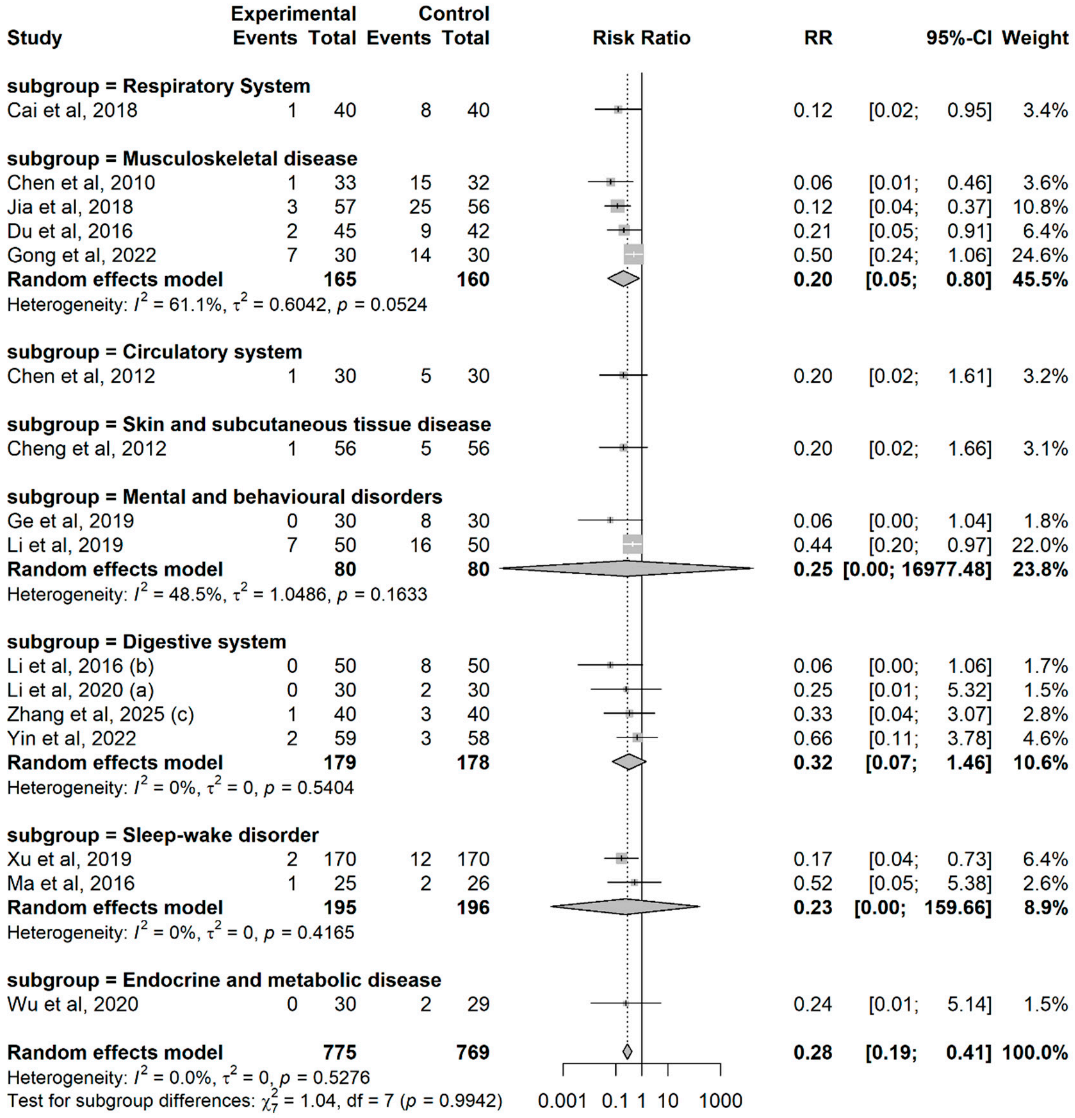

Figure S10. Forest Plot of the Sensitivity Analysis of Overall AEs in Add-on Therapy CM [101,105,108,109,114].

A. Risk Ratio

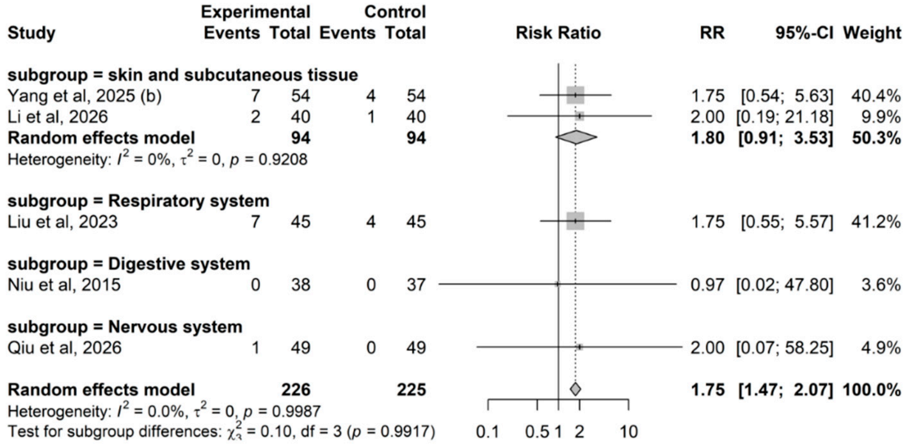

B. Risk difference

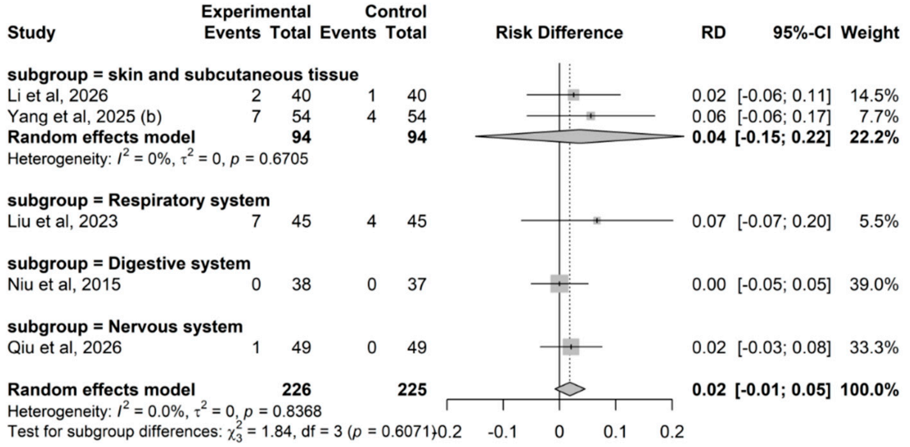

Figure S11. Summary of risk of bias.

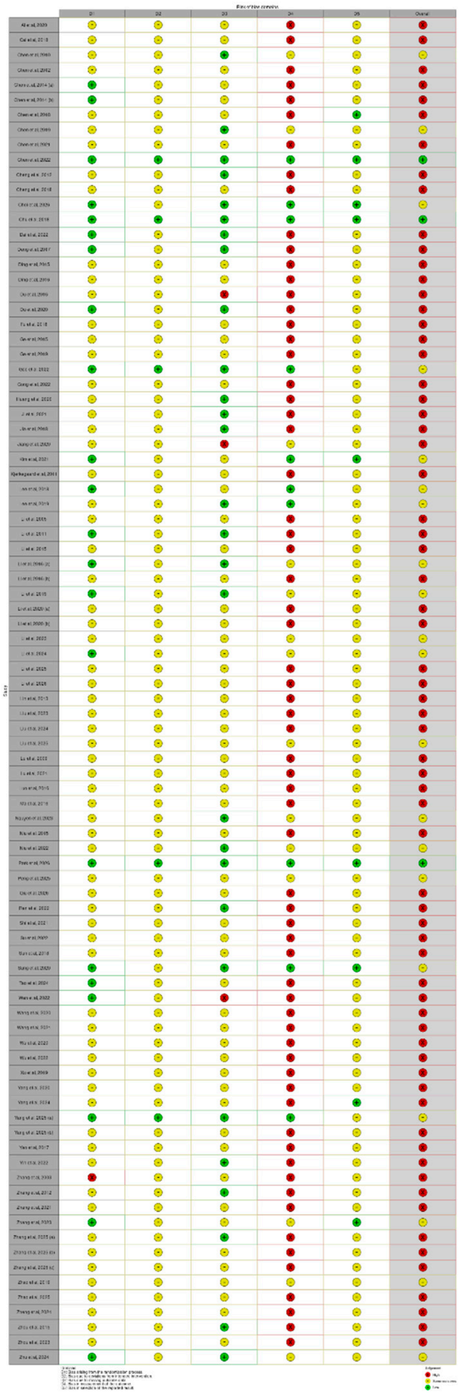

Supplement: Supplementary file 1 [file biomedicines-14-01901-s001.zip › biomedicines-4445149-supplementary.pdf]
